# Supplementary material for: Forest loss and uncertain economic gains from industrial and garimpo mining in Brazilian municipalities
Source: Nat Commun. 2025 Jul 16;16:6543. doi: 10.1038/s41467-025-61930-8 (PMC12267543; doi:10.1038/s41467-025-61930-8)
Supplement: Supplementary file 1 — Supplementary Information [file 41467_2025_61930_MOESM1_ESM.pdf]

Supplementary Information

Forest loss and uncertain economic gains from industrial and garimpo mining in Brazilian municipalities

Sebastian Luckeneder*, Victor Maus, Juliana Siqueira-Gay, Tamás Krisztin, Michael Kuhn

*Correspondence to sebastian.luckeneder@wu.ac.at, Welthandelsplatz 1, 1020 Vienna, Austria

The PDF file includes:

Supplementary Notes A to D

Supplementary Figures S1 to S11

Supplementary Tables S1 to S16

Supplementary Notes

A The Brazilian mining sector

Mining activities vary in size of operation, workforce and their degree of mechanisation. In Brazil, mining titles are issued to companies, cooperatives and individuals by the National Mining Agency as part of the licensing procedures for mining initiatives. Thereby, the Brazilian mining legislation distinguishes between concessions for large-scale commercial mines and the Garimpeira Mining Permission [1]. Originally designed in 1989 to accommodate artisanal and small-scale alluvial mining, this permission sought to recognise and protect miners using rudimentary tools. However, mining techniques within this sector have evolved, albeit often retaining a lower degree of mechanisation, relying on a less specialised workforce and lacking permanent infrastructure. Despite these changes, the regulatory framework for the Garimpeira Mining Permission remains relatively relaxed and decentralised compared to the industrial mining concessions, facilitating "industrial or near-industrial-scale mineral exploration under a weaker regulatory framework"[2]. Showing exceptional expansion since 2008, garimpos today constitute a sector of comparable scale to the highly industrialised commercial mining sector in terms of land utilisation [3]. These operations, primarily focused on gold mining, frequently operate outside legal boundaries, resulting in significant socio-environmental ramifications [1].

Brazil's centre of industrial mining is the state of Minas Gerais, where 145 municipalities (17% of the state) were subject to mining activities in 2020. However, the intensification of mining has mostly occurred in the Legal Amazon area, where huge industrial mining projects such as the Carajás iron ore complex or the Paragominas bauxite mine as well as garimpo mining activities along Amazonian river banks [4] have expanded. The surge in mining in the Amazon – in 2020, 68 out of every 100 ha mined in Brazil – sug-

gests a shift in the socioeconomic and environmental characteristics of mining areas, such as increased disturbance of pristine forest ecosystems and indigenous communities in the Legal Amazon. Fig. S1 depicts how mining area was distributed across Brazilian biomes in 2005, 2010, 2015 and 2020.

Having its economy firmly oriented towards the export of natural resources, Brazil has experienced significant economic ups and downs in the past two decades. Economic expansion took place between 2000 and 2011, and it is no coincidence that global commodity prices were rising during most of that time, which is known as the 2000s commodities boom. However, after having recovered from the global financial crisis, commodity prices fell steadily between 2011 and 2016. A decrease in demand, especially from the Chinese market, and falling mining revenues led to a deep recession in 2014.

B Spatial weights matrix

Spatial econometric models operationalise spatial dependence using weights matrices. These are in most cases constructed by exploiting information on geographic contiguity or distance between spatial units (see, e.g., [5] for further information on spatial weights matrices). In this study, we used a $k = 5$ nearest neighbours specification as illustrated in Fig. S4, defining the neighbours of a municipality as the five closest spatial units next to this municipality (measured at municipality centroids). In a fist step, neighbourhood was defined as a binary indicator. Subsequently, the matrix was row-standardized by dividing each cell of the matrix (either a 0 or a 1) by its respective row sum, such that the entries of each row add up to one. The specification implies positive entries in the matrix for neighbouring observations, but also that the matrix is not symmetric. The spatial weights matrix was computed and visualised using the `sf` [6] and `spdep` [7] packages in R [8].

It is not our intention to capture the exact spatial relations between Brazilian municipalities (it would be impossible to come up with such a measure), but to model spatial dependence in a realistic yet parsimonious way. We are aware that the row-standardization implies that the spatial lag ($\mathbf{Wy}$ and$\mathbf{WX}$) is a weighted average of observations, which is a simplification of spatial dependence [9], but the transformation ensures the stability condition that the spatial parameter is bound between -1 and 1 [10]. Furthermore, while there are other, in some sense less simplifying, approaches in defining neighbourhood such as trade and investment links or shared characteristics such as languages instead of geographic distance, we stick to using geographic proximity as a proxy for connectivity. Most non-geographic connectivity specifications are rather suitable for cross-country studies or demand complex municipality-level trade models due to patchy municipality-level flow data. Due to the strong heterogeneity regarding municipality size, we preferred a k-nearest neighbours specification over a contiguity or inverse distance specification. As shown in Figs. S8 and S9, impact estimates are robust against alternative $k = 4$, 7, and 10 nearest neighbours definitions. However, the spatial parameter ρ varied with the choice of k. For industrial mining, we observed ρ values of 0.26, 0.34, and 0.40 in the economic growth model; 0.57, 0.66, and 0.70 in the relative forest loss model; and 0.51, 0.60, and 0.66 in the absolute forest loss model. For garimpo mining, ρ values were 0.23, 0.30, and 0.35 in the economic growth model; 0.62, 0.70, and 0.74 in the relative forest loss model; and 0.42, 0.49, and 0.54 in the absolute forest loss model.

C MCMC estimation

We used Bayesian Markov-chain Monte Carlo (MCMC) techniques in order to estimate the unknown parameters of the spatial Durbin models (SDM). The interested reader is referred to [10] and [11] for more information on estimating Bayesian spatial econometric

models. Weakly informative multivariate Gaussian priors, centred on zero with a large variance of 10^4, were used for the parameters β, θ, δ and γ. The disturbance parameters σ^2 and $\tilde{\sigma}^2$ were drawn from inverse Gamma distributions $IG(0.01, 0.01)$ using weakly influential shape and scale parameters. For the spatial autocorrelation parameters ρ and λ, we used prior distributions defined on the interval $(-1, 1)$ and centred on zero as suggested in [12]:

$$\rho, \lambda \sim \frac{1}{Beta(a_0, a_0)} \frac{(1+\rho)^{a_o-1}(1-\rho)^{a_o-1}}{2^{2a_0-1}} \tag{1}$$

with hyperparameter value $a_0 = 1.01$.

The conditional posterior distributions for the parameters β, θ, δ, γ, σ^2 and $\tilde{\sigma}^2$ follow known forms and were obtained using standard Gibbs sampling. The conditional distributions of the spatial parameters, however, are not reducible to well-known distributions. We thus used the Griddy Gibbs approach proposed by Ritter and Tanner [13] in order to sample for ρ and λ. MCMC estimation results were obtained from 20,000 iterations and discarding the first 10,000 as burn-ins. Models ran for robustness checks were obtained from 2,000 iterations discarding the first 1,000. Estimations were performed in R [8]. The diagnostics by Geweke [14] were used to confirm convergence of the sampler using the `coda` [15] R package.

D Regression results

Economic growth models

Beyond mining-related effects, the economic growth models reveal associations between land cover transitions and municipal economic growth rates. Tables S5 and S6 present results for the industrial mining sample (LUC control variables), while Tables S11 and S12 report findings for garimpo mining.

The effects of initial land cover classifications – reflecting municipal characteristics at

the start of a 5-year growth window – can further be interpreted. The results indicate a positive direct effect of agriculture in both the industrial and garimpo mining samples. Additionally, agricultural land, forest plantations, and pasture were, for instance, associated with negative indirect effects in the garimpo sample.

The growth models include additional control variables, summarised as follows: We find significant evidence of growth convergence, indicated by negative direct effects of initial income that outweigh positive spillover effects from high-income neighbours. This aligns with theoretical and empirical growth literature [16, 17]. For human capital, effects were in opposite direction, again conforming to earlier works [17, 18]. Population growth was negatively associated with economic growth in both the industrial and garimpo mining samples. In the garimpo sample, spillover effects of population growth were also slightly negative, whereas population density was positively associated with spillover effects. Regarding sectoral structure, initial GVA in the service sector was positively associated with economic growth, while municipalities with larger shares in the industrial sector tended to exhibit lower growth rates. Lastly, we examine ρ, the spatial parameter, which indicates spatial dependence, with estimates of 0.29 in the industrial mining sample and 0.26 in the garimpo sample.

Forest loss models

Regardless of whether forest loss is defined as hectares per km^2 (Tables S7 and S8 for industrial mining; Tables S13 and S14 for garimpo) or in absolute terms (Tables S9 and S10 for industrial mining; Tables S15 and S16 for garimpo), initial natural forest area was consistently associated with forest loss in the respective municipalities. Spillover associations were positive in the industrial mining sample and negative in the garimpo sample. Associations for other land cover and land cover change variables were less

conclusive.

Turning to the remaining control variables, results indicate that GDP growth was associated with spillover effects on forest loss in the garimpo sample, while no such associations were found for the industrial mining subset after matching. Biophysical variables, including precipitation and elevation, played a minor role, except for the observation that municipalities at higher altitudes experienced lower forest loss. Lastly, the strong spatial dependence of forest loss ($\rho = 0.61$ and $\rho = 0.65$ for ha per km^2; $\rho = 0.55$ and $\rho = 0.42$ for absolute ha) underscores the necessity of spatial regression approaches.

Supplementary Figures S1 to S11

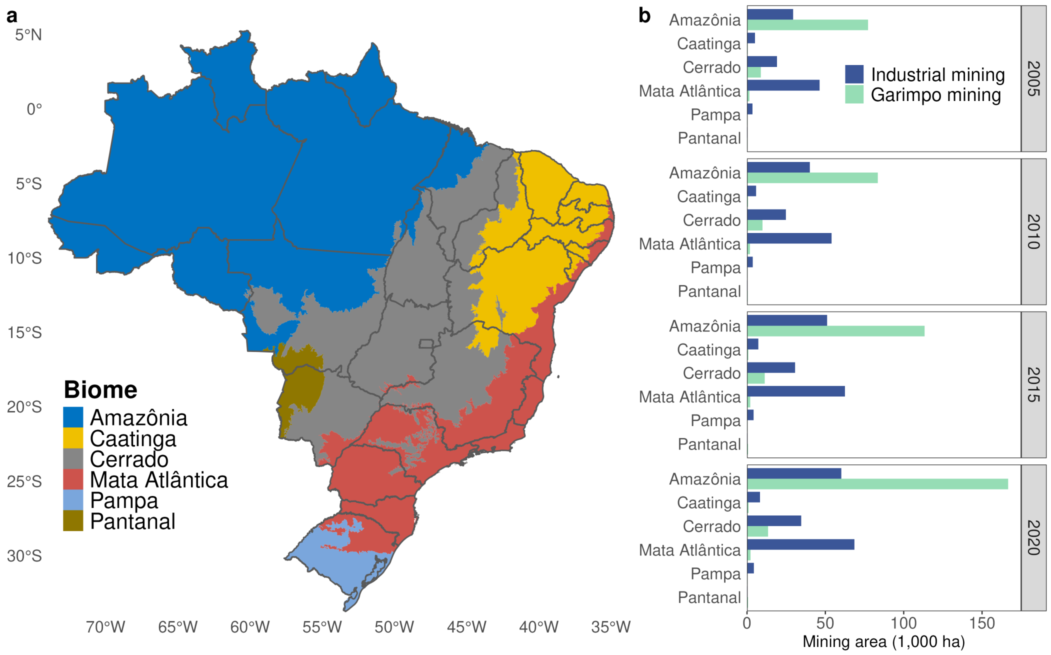

Figure S1: **Brazilian biomes and mining area.** The six terrestrial biomes of Brazil and state borders **(a)**. Mining area (in 1,000 ha) within Brazilian biomes in 2005, 2010, 2015 and 2020 **(b)**. Mining area data from MapBiomas [19], released under a CC Attribution-ShareAlike 4.0 International Licence (CC BY-SA 4.0). Basemaps from the IBGE, made available under a CC-BY 4.0 license [20], accessed via the `geobr` R package [21].

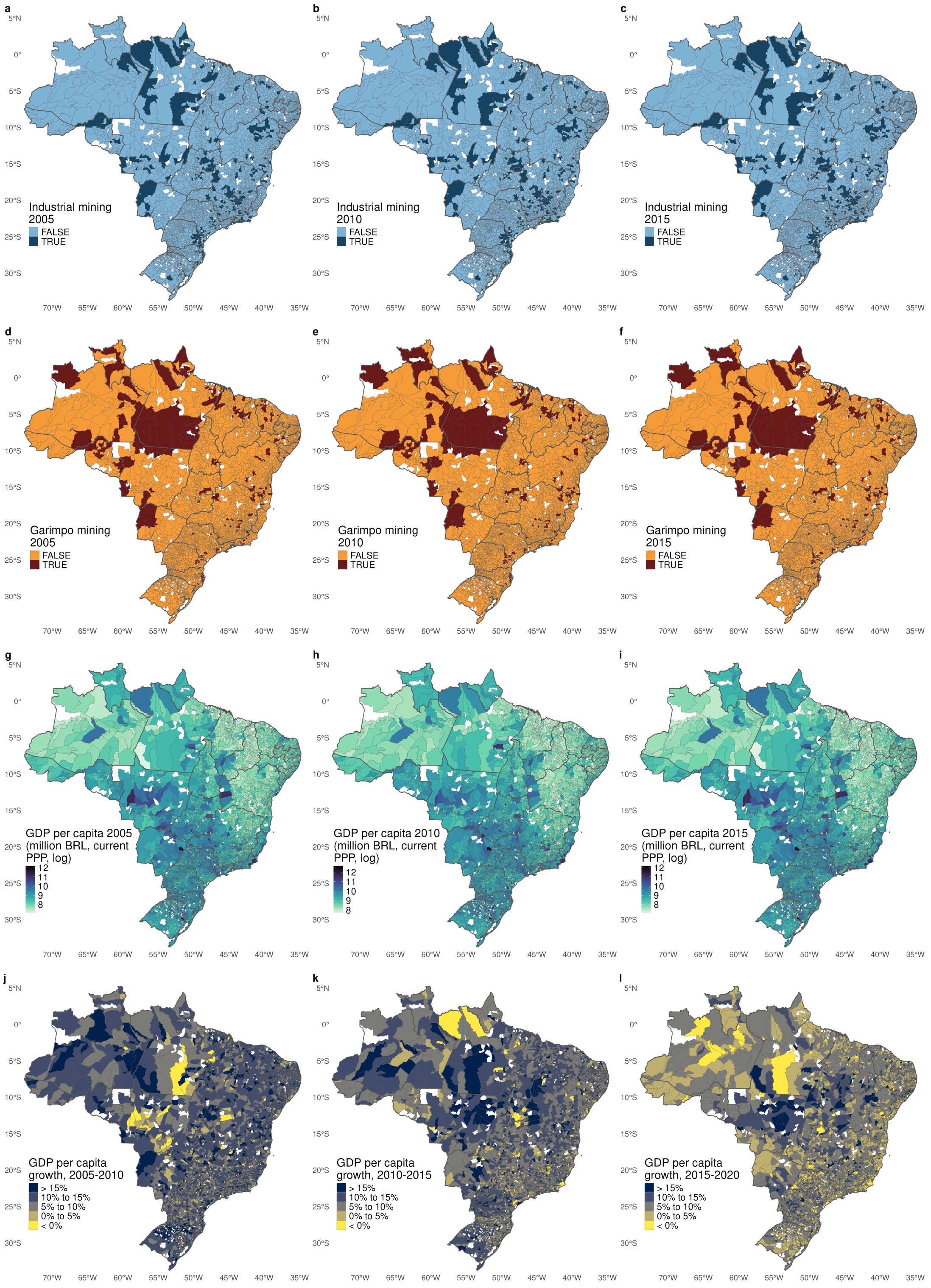

Figure S2: **Selection of variables used in the analysis (2005, 2010, 2015).** Industrial mining binary indicator **(a-c)**, garimpo mining binary indicator **(d-f)**, GDP per capita **(g-i)** and GDP per capita 5-year average annual growth rates **(j-l)**. White areas were not considered in the study due to changes of municipality borders during the sample period. See Table S1 for data sources. Basemaps from the IBGE, made available under a CC-BY 4.0 license [20], accessed via the `geobr` R package [21].

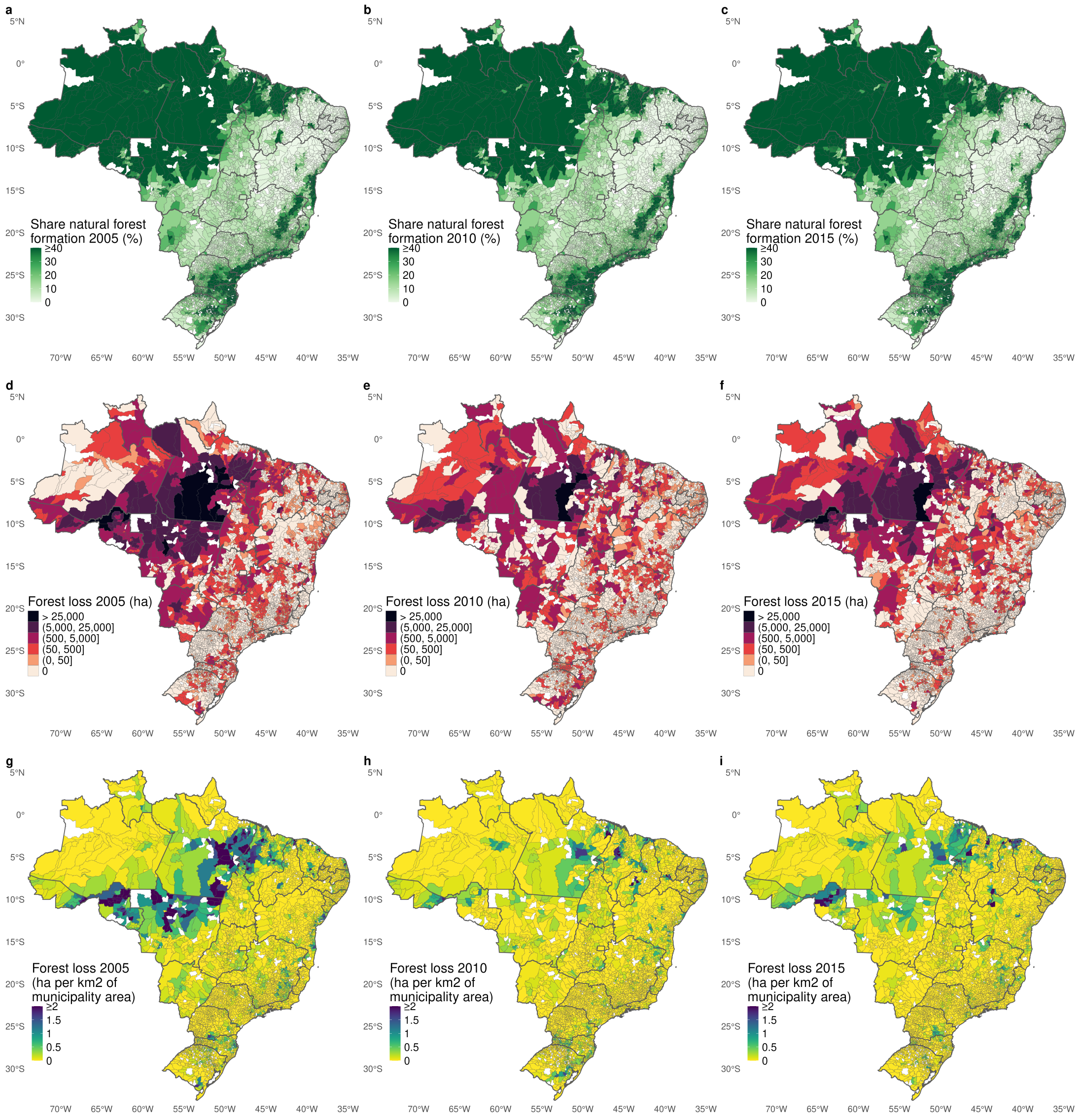

Figure S3: **Selection of variables used in the analysis (2005, 2010, 2015).** Share of area covered by natural forest formation **(a-c)**, absolute forest loss, i.e. decrease in natural forest cover in ha **(d-f)** and relative forest loss, i.e. decrease in natural forest cover relative to municipality area in ha per km^2 **(g-i)**. White areas were not considered in the study due to changes of municipality borders during the sample period. See Table S1 for data sources. Basemaps from the IBGE, made available under a CC-BY 4.0 license [20], accessed via the `geobr` R package [21].

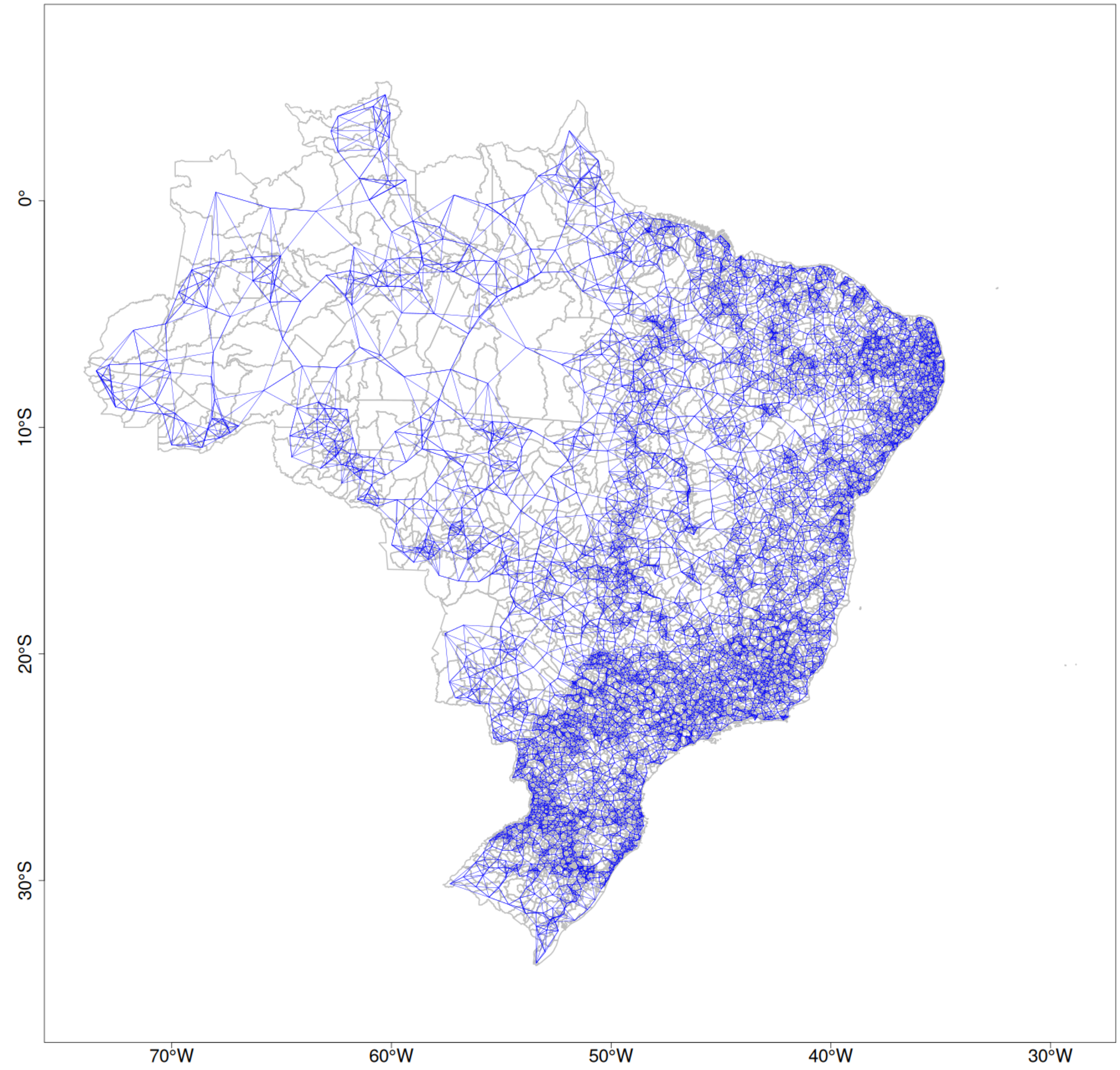

Figure S4: **Spatial weights matrix.** Visualisation of the $k = 5$ nearest neighbours spatial weights matrix employed in this study. Blue lines indicate neighbourhood, grey lines show municipality borders. Basemap from the IBGE, made available under a CC-BY 4.0 license [20], accessed via the `geobr` R package [21].

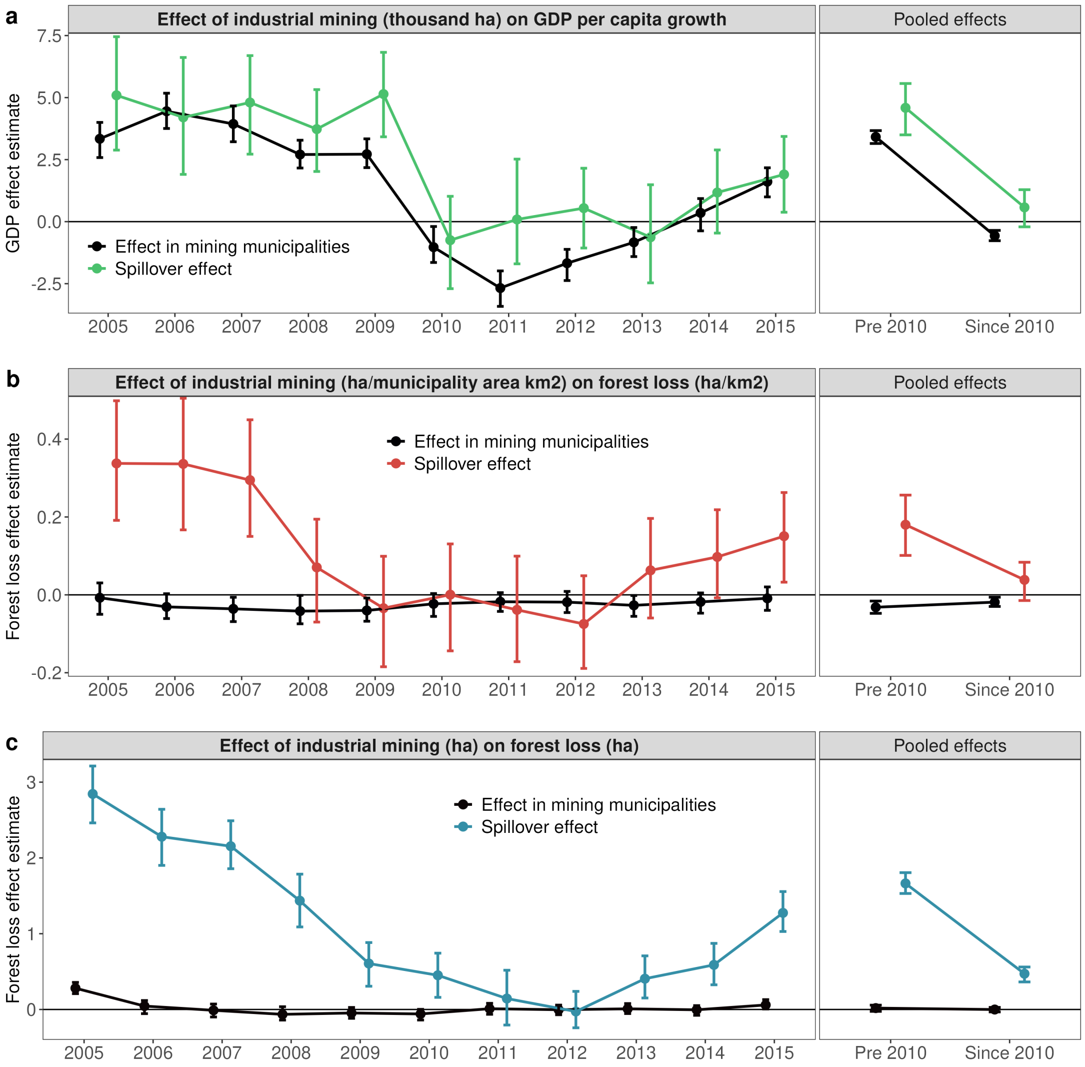

Figure S5: **Direct and spillover effect estimates of industrial mining.** Effects of industrial mining measured in ha on 5-year average annual GDP per capita growth **(a)**, forest loss in ha per km^2 **(b)** and forest loss in ha **(c)**. Left panels show yearly estimates, right panels show pooled (pre 2010 and since 2010) estimates. Estimates were obtained from 2,000 Markov chain Monte Carlo iterations, with the first 1,000 being discarded as burn-in. Points denote posterior means, error bars show 95% posterior credible intervals.

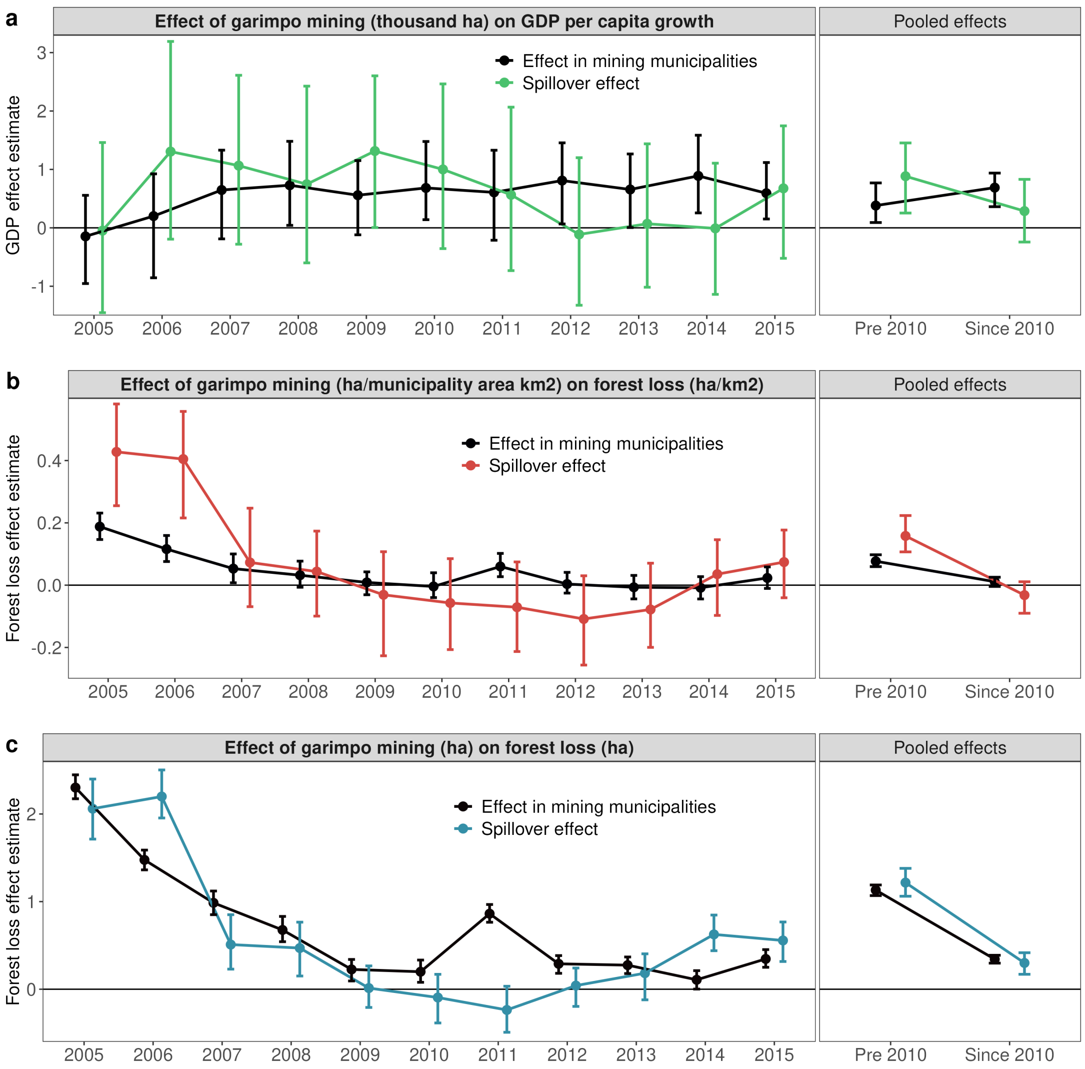

Figure S6: **Direct and spillover effect estimates of garimpo mining.** Effects of garimpo mining measured in ha on 5-year average annual GDP per capita growth **(a)**, forest loss in ha per km^2 **(b)** and forest loss in ha **(c)**. Left panels show yearly estimates, right panels show pooled (pre 2010 and since 2010) estimates. Estimates were obtained from 2,000 Markov chain Monte Carlo iterations, with the first 1,000 being discarded as burn-in. Points denote posterior means, error bars show 95% posterior credible intervals.

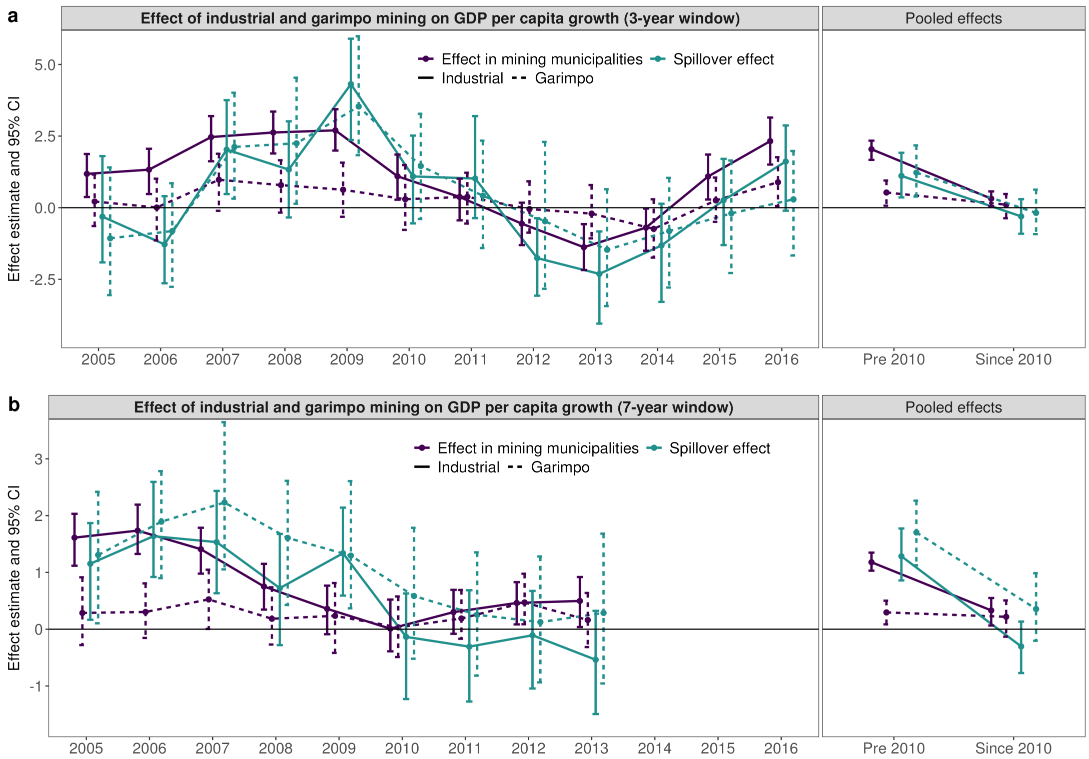

Figure S7: **Direct and spillover effect estimates.** Effects of binary mining indicator on 3-year average GDP per capita growth **(a)** and 7-year average GDP per capita growth **(b)**. Left panels show yearly estimates, right panels show pooled (pre 2010 and since 2010) estimates. Estimates were obtained from 2,000 Markov chain Monte Carlo iterations, with the first 1,000 being discarded as burn-in. Points denote posterior means, error bars show 95% posterior credible intervals.

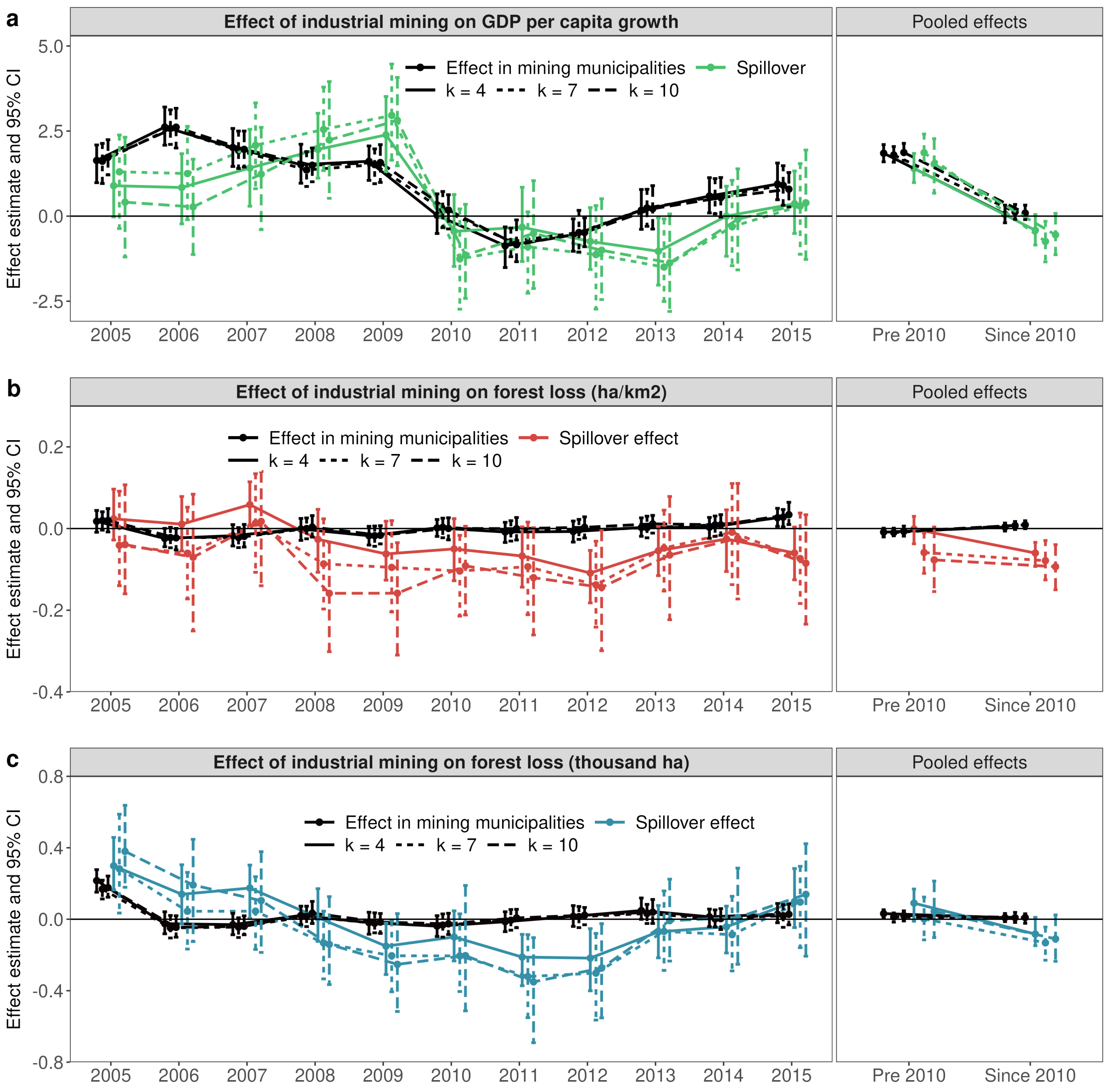

Figure S8: **Direct and spillover effect estimates of industrial mining.** Effects of binary industrial mining indicator on 5-year average GDP per capita growth **(a)**, forest loss in ha per km^2 **(b)** and forest loss in thousand ha **(c)**. Left panels show yearly estimates, right panels show pooled (pre 2010 and since 2010) estimates. Different linetypes show results using $k = 4$, $k = 7$ and $k = 10$ nearest neighbours spatial weights matrices. Estimates were obtained from 2,000 Markov chain Monte Carlo iterations, with the first 1,000 being discarded as burn-in. Points denote posterior means, error bars show 95% posterior credible intervals.

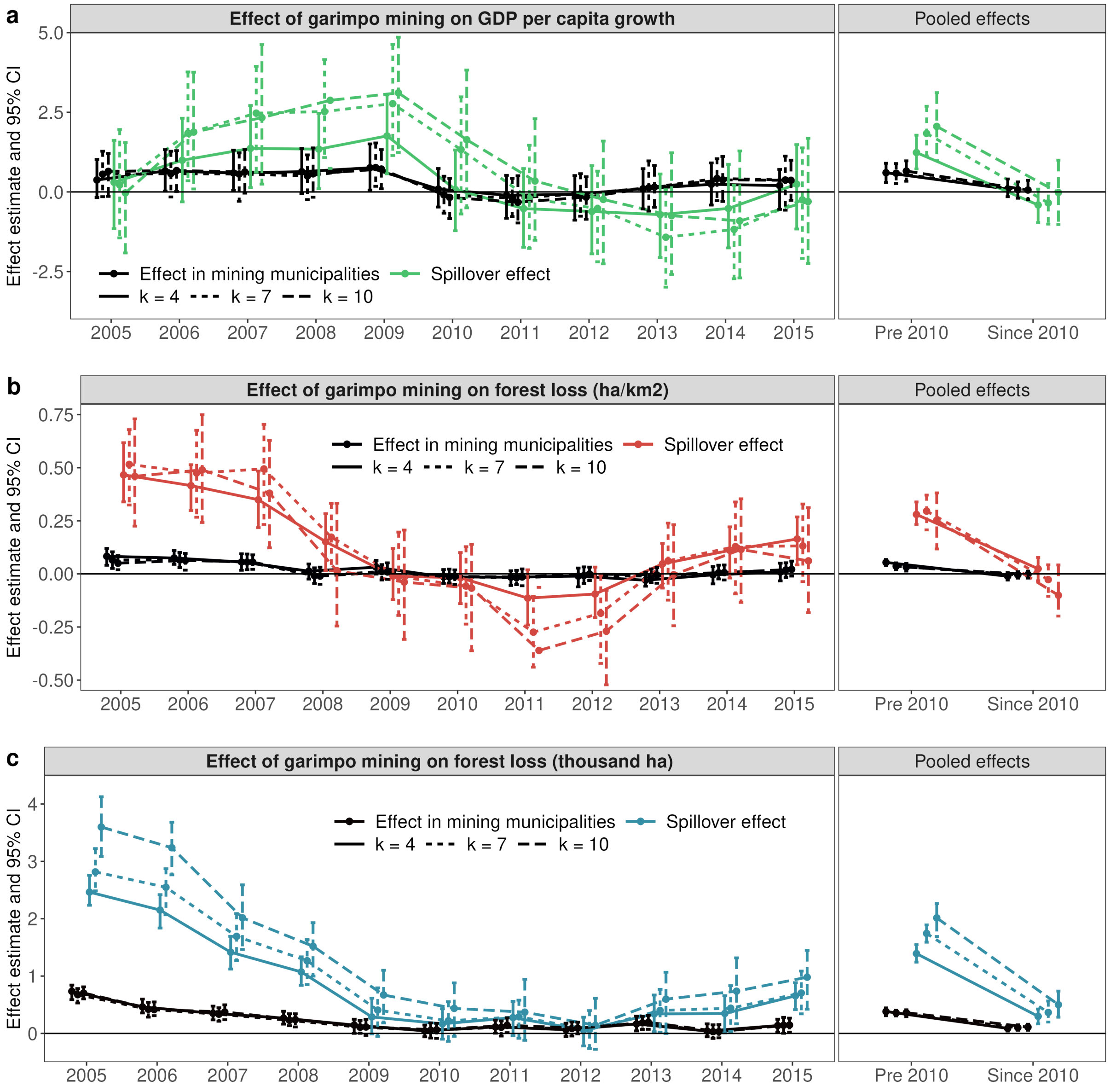

Figure S9: **Direct and spillover effect estimates of garimpo mining.** Effects of binary garimpo mining indicator on 5-year average GDP per capita growth **(a)**, forest loss in ha per km^2 **(b)** and forest loss in thousand ha **(c)**. Left panels show yearly estimates, right panels show pooled (pre 2010 and since 2010) estimates. Different linetypes show results using $k = 4$, $k = 7$ and $k = 10$ nearest neighbours spatial weights matrices. Estimates were obtained from 2,000 Markov chain Monte Carlo iterations, with the first 1,000 being discarded as burn-in. Points denote posterior means, error bars show 95% posterior credible intervals.

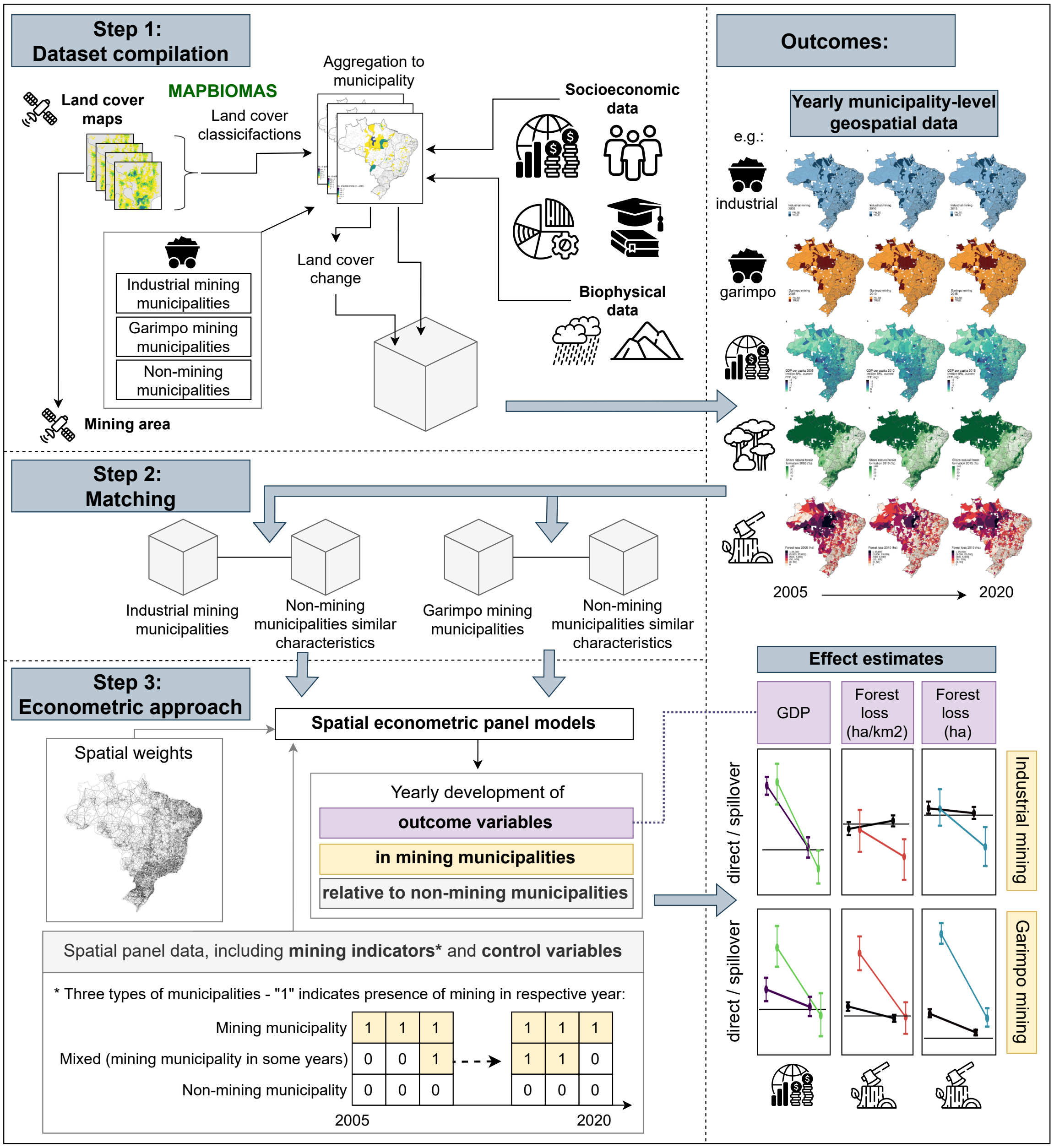

Figure S10: **Overview of the analytical framework.** The workflow consists of three main steps: (1) dataset compilation, (2) the application of coarsened exact matching to prune the compiled data, and (3) a spatial econometric approach applied separately to industrial and garimpo mining. Each matched dataset is used to estimate effects on three outcome variables: municipality-level per capita GDP, relative forest loss (ha/km^2), and absolute forest loss (ha). The final outputs are the effect estimates derived from the spatial models. Icons used in this figure were designed by Freepik (www.freepik.com) and the users surang, AA GRAPHICS, Dewi Sari, irakun08, rananjay1, Georgiana_Lavinia and hillvector.

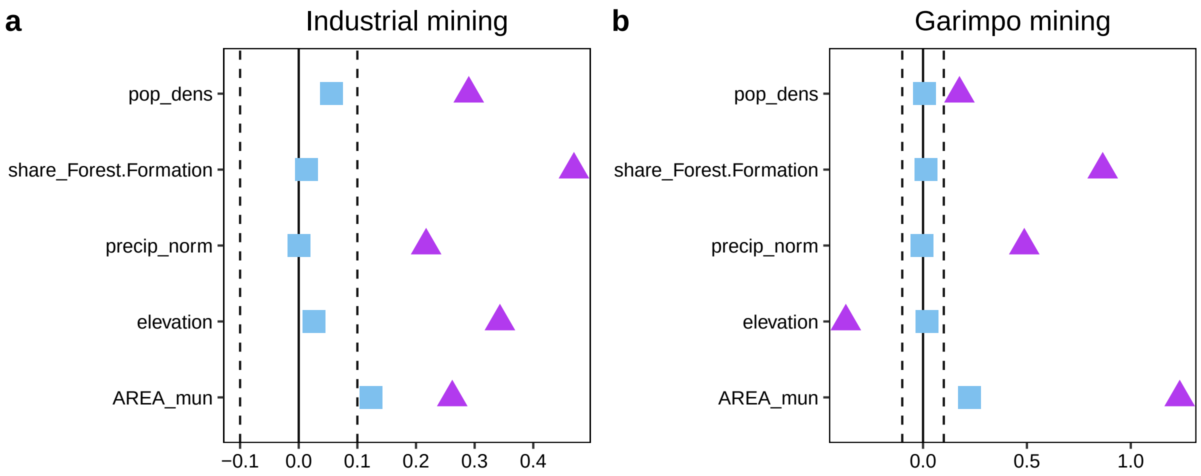

Figure S11: **Standardised mean differences (SMD) between mining and non-mining observations.** SMD for matching covariates before (triangle) and after (square) matching for industrial **(a)** and garimpo mining **(b)**. Dashed lines indicate a threshold of 0.1.

Tables S1 to S16

Variable	Description	GM	FLM
Economic growth	Five-year average annual growth rate of gross domestic product per capita. *Source:* IBGE [22, 23]	D	I
Forest loss (relative)	Annual decrease in natural forest formation relative to municipality area (ha/km^2). *Source:* MapBiomas [19]		D
Forest loss (absolute)	Annual decrease in natural forest formation (ha). *Source:* MapBiomas [19]		D
Industrial mining	Presence of industrial mining within municipality, binary indicator. *Source:* MapBiomas [19]	I	I
Garimpo mining	Presence of garimpo mining within municipality, binary indicator. *Source:* MapBiomas [19]	I	I
Land use change (LUC^{1,2})	Land use change from classification LUC^1 to LUC^2 for the classifications forest formation, forest plantation, grassland, agriculture and pasture (5-year average change in ha, log). *Source:* MapBiomas [19]	I	I
Initial natural forest	Share classified as forest formation. *Source:* MapBiomas [19]	I	I
Initial forest plantation	Share classified as forest plantation. *Source:* MapBiomas [19]	I	I
Initial grassland	Share classified as grassland. *Source:* MapBiomas [19]	I	I
Initial agriculture	Share classified as agriculture. *Source:* MapBiomas [19]	I	I
Initial pasture	Share classified as pasture. *Source:* MapBiomas [19]	I	I
Initial income	Per capita gross domestic product (million BRL, current PPP, log). *Source:* IBGE [22, 23]	I	
Human capital	Education index from 0 (worst) to 1 (best): schooling coverage (pre-school attendance) and quality in elementary school. *Source:* FIRJAN [24]	I	
Population growth	Population growth rate (%). *Source:* IBGE [22]	I	
Population density	Population density (thousand per km^2). *Source:* IBGE [22]	I	
GVA agriculture	Gross value added in agriculture (million BRL, current PPP, log). *Source:* IBGE [23]	I	
GVA industry	Gross value added in industry (million BRL, current PPP, log). *Source:* IBGE [23]	I	
GVA services	Gross value added in services (million BRL, current PPP, log). *Source:* IBGE [23]	I	
Precipitation	Precipitation yearly average (standardised). *Source:* CRU [25]	I	I
Elevation	Average elevation (m). *Source:* USGS [26]	I	I

Table S1: **Variables used in the analysis** (measured at the beginning of the respective growth/forest loss window). GM and FLM indicate use of variables in growth and forest loss models, respectively. D denotes dependent and I denotes independent variables. Variables are mapped in Figs. S2 and S3.

Covariate group	Considered variable*	Rationale
Mining	Industrial mining	First key covariate in the GDP models, expected to influence local GDP through employment, local procurement, economic multipliers, population growth, and potential resource curse effects [27–35].
	Garimpo mining	Second key covariate in GDP models, potentially influencing local GDP through similar mechanisms as industrial mining but in a different way due to its smaller scale and informal, often illegal nature [1, 27–35].
Local environmental conditions	Initial natural forest	Represents accessibility and suitability for land development [35]. Accessibility and land competition may, in turn, influence mine allocation.
	Initial grassland	See initial natural forest.
	Precipitation	See initial natural forest.
	Elevation	See initial natural forest.
Local economic conditions	Initial forest plantation	Associated with employment and income effects, economic multipliers, and population pull dynamics. Land competition may, in turn, influence the allocation of mining activities.
	Initial agriculture	See initial forest plantation.
	Initial pasture	See initial forest plantation.
	Initial income	Captures growth convergence, a stylised fact from neoclassical growth theory explained by diminishing returns to capital accumulation [36], as well as agglomeration effects [16]. May correlate with energy and transport infrastructure, thereby facilitating mine development; but also with competing land uses or planning restrictions, which may hinder mine allocation.
	GVA agriculture	Reflects economic structure and sectoral contributions to local economic activity. Sectoral GVA contributes to GDP growth through productivity gains, employment, and income generation, with varying growth effects depending on sectoral linkages [18]. May also facilitate or constrain mine development, depending on whether land use supports coexistence or creates competition.
	GVA industry	See GVA agriculture.
	GVA services	See GVA agriculture.
Local sociodemagraphic conditions	Human capital	Schooling coverage and quality drives human capital development, fostering innovation [37, 38], while potentially contributing to brain drain. May influence local mine development; industrial mining may benefit from skilled labour, while garimpo mining is less reliant on human capital.
	Population growth	Influences local labour supply and demand [17].
	Population density	Facilitates agglomeration effects, driving productivity growth through economies of scale and knowledge spillovers [17]. May hinder mine development due to environmental and social regulatory pressures.
Changes in local environmental and economic conditions	Land use change	Captures shifts between land use types (e.g., grassland to pasture or agriculture), influencing GDP through changes in resource use, employment, and sectoral productivity, while highlighting economy-environment trade-offs.

Table S2: **Covariates influencing municipality GDP per capita growth and mine allocation.** Considered variables and their rationale for inclusion. *Refer to Table S1 for variable descriptions and sources.

Covariate group	Considered variable*	Rationale
Mining	Industrial mining	First key covariate in the forest loss models, expected to influence forest cover through, mine expansion, transport and energy infrastructure development, population pull effects, and associated urban and agricultural expansion [35, 39, 40].
	Garimpo mining	Second key covariate in forest loss models, potentially influencing forest cover through similar mechanisms as industrial mining but in a different way due to its smaller scale and informal, often illegal nature [1, 35, 39, 40].
Local environmental conditions	Initial natural forest	A prerequisite for forest loss, as greater initial forest cover increases the potential for deforestation [35, 41, 42]. Also serves as an indicator of accessibility and suitability for land development, which may influence mine allocation.
	Initial grassland	Expected to reduce potential forest loss, as greater grassland cover limits the area available for deforestation. Also serves as an indicator of accessibility and suitability for land development, which may, in turn, influence mine allocation.
	Precipitation	Affects forest cover by supporting forest growth and influencing land development suitability, while also lowering accessibility. Extreme rainfall or drought can increase vulnerability to wildfires, erosion, and other factors that promote deforestation [35, 41, 42]. Accessibility and land competition may, in turn, influence mine allocation.
	Elevation	Influences forest loss by affecting climate, vegetation, and land development. Higher elevations, with cooler temperatures and lower settlement, may reduce deforestation potential, while lower elevations, being more accessible, face greater pressure from agricultural expansion and infrastructure development [35]. Accessibility and land competition may, in turn, influence mine allocation.
Local economic conditions	Initial forest plantation	Influences forest loss by determining the extent of land available for conversion to other uses. Land competition may, in turn, influence mine allocation.
	Initial agriculture	Established agricultural areas reduce the potential for further deforestation, but may also drive expansion into adjacent forests [41, 42]. Land competition may, in turn, influence mine allocation.
	Initial pasture	See initial agriculture.
Changes in local environmental and economic conditions	Economic growth	Reflects overall economic activity, which can drive forest loss through increased demand for land, resources, and infrastructure, often leading to expanded agriculture, urbanisation, and industry [41, 42]. May correlate with energy and transport infrastructure, thereby facilitating mine development; but also with competing land uses or planning restrictions, which may hinder mine allocation.
	Land use change	Transformations from non-forest areas (e.g., grassland) to agriculture or pasture may increase pressure on surrounding forests, driving further deforestation [41, 42].

Table S3: **Covariates influencing forest cover change and mine allocation.** Considered variables and their rationale for inclusion. *Refer to Table S1 for variable descriptions and sources.

	Before matching			Matched industrial		Matched garimpo	
Variable	Industrial	Garimpo	Non-mining	Industrial	Non-mining	Garimpo	Non-mining
Initial agriculture	0.05	0.03	0.13	0.06	0.13	0.03	0.12
Initial natural forest	0.28	0.36	0.18	0.27	0.15	0.33	0.15
Initial forest plantation	0.02	0.01	0.01	0.02	0.01	0.01	0.01
Initial grassland	0.03	0.03	0.02	0.03	0.02	0.03	0.02
Initial pasture	0.30	0.29	0.31	0.32	0.34	0.31	0.34
Initial income	9.48	9.14	9.10	9.46	9.08	9.11	9.06
Human capital	0.69	0.62	0.68	0.69	0.68	0.63	0.67
Population growth	1.13	1.17	0.60	1.11	0.60	1.13	0.66
Population density	0.27	0.21	0.10	0.15	0.05	0.10	0.06
GVA agriculture	2.79	3.06	2.66	2.84	2.66	3.01	2.63
GVA industry	4.30	3.57	2.13	4.11	2.08	3.38	2.14
GVA services	4.87	4.46	3.33	4.68	3.29	4.28	3.33
Precipitation	0.17	0.42	-0.06	0.15	-0.15	0.30	-0.22
Elevation	543.20	353.40	451.19	543.43	442.26	369.70	415.64
Area	2807.43	6589.72	1256.83	1695.15	907.93	2982.36	960.83
Observations	4,430	2,753	51,707	3,923	42,646	2,356	35,156

Table S4: **Covariate balance between mining and non-mining municipalities before and after matching.** Average values for industrial-, garimpo- and non-mining municipalities before and after the matching procedure. For the corresponding standardised mean differences of the matching covariates, see Fig. S11.

Variables	Avg. direct effect 2.5%	PM	97.5%	Avg. spillover 2.5%	PM	97.5%
Industrial mining 2005	1.016	**1.577**	2.133	0.043	**1.207**	2.350
Industrial mining 2006	1.919	**2.482**	3.064	-0.061	1.000	2.108
Industrial mining 2007	1.328	**1.900**	2.480	0.370	**1.584**	2.712
Industrial mining 2008	0.901	**1.424**	1.929	0.832	**1.987**	3.150
Industrial mining 2009	0.973	**1.502**	2.064	1.393	**2.516**	3.548
Industrial mining 2010	-0.479	0.120	0.675	-1.866	-0.766	0.357
Industrial mining 2011	-1.379	**-0.807**	-0.266	-1.616	-0.507	0.600
Industrial mining 2012	-1.055	-0.513	0.091	-1.999	-0.816	0.394
Industrial mining 2013	-0.320	0.218	0.802	-2.301	**-1.175**	-0.044
Industrial mining 2014	0.061	**0.585**	1.145	-1.284	-0.129	1.036
Industrial mining 2015	0.334	**0.884**	1.437	-0.888	0.225	1.311
$LUC^{Agriculture,ForestPlantation}$	0.029	**0.117**	0.204	-0.101	0.109	0.318
$LUC^{Agriculture,Grassland}$	-0.146	-0.040	0.063	0.325	**0.607**	0.882
$LUC^{Agriculture,Pasture}$	-0.056	-0.017	0.024	0.035	**0.122**	0.210
$LUC^{NaturalForest,Agriculture}$	-0.058	0.051	0.163	-0.179	0.104	0.374
$LUC^{NaturalForest,ForestPlantation}$	-0.017	0.038	0.094	-0.224	-0.099	0.025
$LUC^{NaturalForest,Grassland}$	-0.092	-0.018	0.060	-0.345	-0.153	0.048
$LUC^{NaturalForest,Pasture}$	-0.050	-0.004	0.041	-0.147	**-0.079**	-0.008
$LUC^{ForestPlantation,Agriculture}$	-0.056	0.065	0.178	-0.584	**-0.299**	-0.009
$LUC^{ForestPlantation,Grassland}$	-0.015	0.115	0.237	-0.486	-0.165	0.131
$LUC^{ForestPlantation,Pasture}$	-0.079	-0.014	0.052	-0.074	0.095	0.254
$LUC^{Grassland,Agriculture}$	-0.003	0.092	0.183	-0.325	-0.102	0.115
$LUC^{Grassland,ForestPlantation}$	-0.165	-0.077	0.009	-0.611	**-0.424**	-0.230
$LUC^{Grassland,Pasture}$	-0.059	-0.007	0.046	0.179	**0.288**	0.405
$LUC^{Pasture,Agriculture}$	0.037	**0.071**	0.105	-0.066	0.008	0.079
$LUC^{Pasture,ForestPlantation}$	-0.040	0.007	0.052	-0.082	0.005	0.095
$LUC^{Pasture,Grassland}$	0.084	**0.136**	0.190	-0.225	**-0.114**	-0.006
Initial agriculture	1.125	**1.837**	2.543	-1.124	-0.112	0.842
Initial natural forest	-1.873	**-1.071**	-0.274	2.434	**3.702**	4.906
Initial forest plantation	-0.377	1.578	3.501	-8.317	**-4.560**	-0.986
Initial grassland	-2.858	**-1.406**	-0.052	1.434	**3.978**	6.390
Initial pasture	-0.669	-0.120	0.461	-1.054	-0.371	0.319
Initial income	-3.033	**-2.878**	-2.730	1.487	**1.783**	2.072
Human capital	1.839	**2.591**	3.342	-2.745	**-1.604**	-0.539
Population growth	-0.452	**-0.420**	-0.388	-0.063	0.015	0.093
Population density	-1.330	-0.487	0.289	-1.002	0.674	2.424
GVA agriculture	-0.109	-0.037	0.030	-0.233	-0.092	0.049
GVA industry	-0.362	**-0.289**	-0.218	-0.741	**-0.578**	-0.399
GVA services	0.366	**0.461**	0.551	0.106	**0.321**	0.531
Precipitation	0.248	**0.750**	1.232	-0.936	-0.449	0.051
Elevation	-0.000	0.001	0.001	-0.002	**-0.001**	-0.000
ρ	0.274	**0.290**	0.303			
Observations	33,836					

Table S5: **Average direct and spillover effect estimates on economic growth with yearly industrial mining effects.** Panel-structure (2005-2020) spatial Durbin model including time fixed effects. Dependent variable is 5-year average annual GDP per capita growth rate. Estimates printed in bold type are statistically different from zero based on the 95 percent posterior credible interval. PM denotes posterior mean. Time-specific intercepts were excluded for more concise summary tables.

Variables	Avg. direct effect 2.5%	PM	97.5%	Avg. spillover 2.5%	PM	97.5%
Industrial mining × pre 2010	1.554	**1.803**	2.050	1.291	**1.898**	2.496
Industrial mining × since 2010	-0.146	0.076	0.306	-1.078	**-0.543**	-0.023
$LUC^{Agriculture,ForestPlantation}$	0.029	**0.115**	0.197	-0.095	0.115	0.339
$LUC^{Agriculture,Grassland}$	-0.157	-0.039	0.073	0.353	**0.637**	0.920
$LUC^{Agriculture,Pasture}$	-0.053	-0.014	0.024	0.037	**0.127**	0.214
$LUC^{NaturalForest,Agriculture}$	-0.054	0.052	0.155	-0.196	0.100	0.398
$LUC^{NaturalForest,ForestPlantation}$	-0.019	0.037	0.092	-0.233	-0.104	0.030
$LUC^{NaturalForest,Grassland}$	-0.097	-0.019	0.058	-0.360	-0.160	0.045
$LUC^{NaturalForest,Pasture}$	-0.052	-0.004	0.042	-0.155	**-0.080**	-0.006
$LUC^{ForestPlantation,Agriculture}$	-0.043	0.073	0.197	-0.637	**-0.305**	-0.010
$LUC^{ForestPlantation,Grassland}$	-0.024	0.108	0.235	-0.514	-0.176	0.164
$LUC^{ForestPlantation,Pasture}$	-0.079	-0.014	0.049	-0.087	0.088	0.264
$LUC^{Grassland,Agriculture}$	0.005	**0.093**	0.188	-0.319	-0.096	0.138
$LUC^{Grassland,ForestPlantation}$	-0.161	-0.080	0.007	-0.653	**-0.457**	-0.245
$LUC^{Grassland,Pasture}$	-0.060	-0.004	0.050	0.190	**0.305**	0.424
$LUC^{Pasture,Agriculture}$	0.038	**0.071**	0.104	-0.061	0.015	0.088
$LUC^{Pasture,ForestPlantation}$	-0.042	0.004	0.049	-0.086	0.002	0.086
$LUC^{Pasture,Grassland}$	0.081	**0.135**	0.189	-0.222	-0.109	0.005
Initial agriculture	1.126	**1.827**	2.555	-1.063	0.044	1.109
Initial natural forest	-1.774	**-1.054**	-0.293	2.703	**3.926**	5.120
Initial forest plantation	-0.382	1.538	3.521	-8.471	**-4.571**	-0.792
Initial grassland	-2.732	**-1.373**	-0.013	1.672	**4.140**	6.822
Initial pasture	-0.632	-0.118	0.378	-1.069	-0.410	0.264
Initial income	-3.021	**-2.864**	-2.709	1.377	**1.695**	2.002
Human capital	1.879	**2.616**	3.383	-2.560	**-1.507**	-0.360
Population growth	-0.456	**-0.421**	-0.386	-0.089	-0.008	0.075
Population density	-1.296	-0.492	0.281	-0.941	0.836	2.816
GVA agriculture	-0.107	-0.036	0.040	-0.225	-0.085	0.068
GVA industry	-0.377	**-0.299**	-0.224	-0.825	**-0.643**	-0.458
GVA services	0.369	**0.467**	0.562	0.146	**0.368**	0.587
Precipitation	0.282	**0.740**	1.204	-0.923	-0.444	0.042
Elevation	0.000	**0.001**	0.001	-0.002	**-0.001**	-0.000
ρ	0.284	**0.291**	0.303			
Observations	33,836					

Table S6: **Average direct and spillover effect estimates on economic growth with pooled industrial mining effects.** Panel-structure (2005-2020) spatial Durbin model including time fixed effects. Dependent variable is 5-year average annual GDP per capita growth rate. Estimates printed in bold type are statistically different from zero based on the 95 percent posterior credible interval. PM denotes posterior mean. Time-specific intercepts were excluded for more concise summary tables.

Variables	Avg. direct effect 2.5%	PM	97.5%	Avg. spillover 2.5%	PM	97.5%
Industrial mining 2005	-0.011	0.014	0.039	-0.079	0.011	0.104
Industrial mining 2006	-0.047	-0.019	0.007	-0.083	0.003	0.094
Industrial mining 2007	-0.042	-0.017	0.009	-0.049	0.042	0.133
Industrial mining 2008	-0.028	-0.002	0.024	-0.138	-0.047	0.045
Industrial mining 2009	-0.043	-0.017	0.010	-0.165	-0.071	0.024
Industrial mining 2010	-0.021	0.003	0.027	-0.167	-0.077	0.013
Industrial mining 2011	-0.033	-0.009	0.014	-0.170	-0.082	0.013
Industrial mining 2012	-0.032	-0.006	0.019	-0.199	**-0.109**	-0.018
Industrial mining 2013	-0.021	0.004	0.030	-0.130	-0.047	0.038
Industrial mining 2014	-0.018	0.006	0.031	-0.112	-0.022	0.069
Industrial mining 2015	0.002	**0.028**	0.054	-0.139	-0.048	0.042
$LUC^{Agriculture,Grassland}$	-0.015	**-0.010**	-0.005	-0.055	**-0.032**	-0.011
$LUC^{Agriculture,Pasture}$	-0.004	**-0.003**	-0.001	-0.020	**-0.014**	-0.007
$LUC^{Grassland,Agriculture}$	-0.002	0.002	0.006	-0.011	0.009	0.027
$LUC^{Grassland,Pasture}$	-0.001	0.001	0.003	-0.006	0.003	0.011
$LUC^{Pasture,Agriculture}$	-0.002	0.000	0.002	0.004	**0.009**	0.015
$LUC^{Pasture,Grassland}$	-0.005	**-0.003**	-0.000	-0.007	0.001	0.010
Initial agriculture	-0.065	**-0.032**	-0.000	-0.170	**-0.117**	-0.058
Initial natural forest	0.318	**0.350**	0.382	0.035	**0.110**	0.185
Initial forest plantation	-0.056	0.026	0.112	-0.474	**-0.232**	-0.007
Initial grassland	-0.037	0.024	0.079	-0.042	0.140	0.321
Initial pasture	-0.015	0.007	0.029	-0.057	-0.022	0.015
GDP growth	-0.001	-0.000	0.000	-0.001	0.001	0.003
Precipitation	-0.007	0.012	0.032	-0.016	0.006	0.028
Elevation	-0.000	**-0.000**	-0.000	-0.000	**-0.000**	-0.000
ρ	0.602	**0.614**	0.622			
Observations	33,836					

Table S7: **Average direct and spillover effect estimates on forest loss (relative) with yearly industrial mining effects.** Panel-structure (2005-2020) spatial Durbin model including time fixed effects. Dependent variable is annual forest loss in ha per km^2. Estimates printed in bold type are statistically different from zero based on the 95 percent posterior credible interval. PM denotes posterior mean. Time-specific intercepts were excluded for more concise summary tables.

Variables	Avg. direct effect 2.5%	PM	97.5%	Avg. spillover 2.5%	PM	97.5%
Industrial mining × pre 2010	-0.021	-0.008	0.003	-0.059	-0.013	0.033
Industrial mining × since 2010	-0.007	0.004	0.016	-0.105	**-0.064**	-0.023
$LUC^{Agriculture,Grassland}$	-0.015	**-0.009**	-0.004	-0.055	**-0.032**	-0.009
$LUC^{Agriculture,Pasture}$	-0.005	**-0.003**	-0.001	-0.020	**-0.014**	-0.007
$LUC^{Grassland,Agriculture}$	-0.002	0.002	0.006	-0.009	0.009	0.027
$LUC^{Grassland,Pasture}$	-0.001	0.001	0.003	-0.006	0.003	0.012
$LUC^{Pasture,Agriculture}$	-0.002	0.000	0.002	0.003	**0.009**	0.015
$LUC^{Pasture,Grassland}$	-0.005	**-0.003**	-0.001	-0.008	0.002	0.010
Initial agriculture	-0.063	**-0.033**	-0.001	-0.172	**-0.114**	-0.059
Initial natural forest	0.319	**0.349**	0.380	0.043	**0.114**	0.195
Initial forest plantation	-0.056	0.026	0.109	-0.470	-0.225	0.032
Initial grassland	-0.031	0.024	0.080	-0.052	0.133	0.301
Initial pasture	-0.015	0.007	0.030	-0.061	-0.022	0.019
GDP growth	-0.001	-0.000	0.000	-0.001	0.001	0.003
Precipitation	-0.008	0.013	0.033	-0.018	0.005	0.028
Elevation	-0.000	**-0.000**	-0.000	-0.000	**-0.000**	-0.000
ρ	0.602	**0.614**	0.622			
Observations	33,836					

Table S8: **Average direct and spillover effect estimates on forest loss (relative) with pooled industrial mining effects.** Panel-structure (2005-2020) spatial Durbin model including time fixed effects. Dependent variable is annual forest loss in ha per km^2. Estimates printed in bold type are statistically different from zero based on the 95 percent posterior credible interval. PM denotes posterior mean. Time-specific intercepts were excluded for more concise summary tables.

Variables	Avg. direct effect 2.5%	PM	97.5%	Avg. spillover 2.5%	PM	97.5%
Industrial mining 2005	112.994	**175.167**	234.527	33.517	**231.421**	433.147
Industrial mining 2006	-92.739	-31.757	31.002	-128.852	66.817	252.061
Industrial mining 2007	-88.310	-30.756	27.483	-86.487	102.503	293.548
Industrial mining 2008	-39.796	22.278	84.082	-279.254	-87.528	105.631
Industrial mining 2009	-77.587	-16.884	42.788	-388.276	**-189.710**	-13.309
Industrial mining 2010	-86.910	-26.537	35.057	-342.004	-168.942	10.654
Industrial mining 2011	-69.202	-8.445	50.396	-429.452	**-249.880**	-61.090
Industrial mining 2012	-49.522	9.446	70.636	-445.509	**-254.486**	-71.294
Industrial mining 2013	-22.868	34.262	94.050	-295.469	-115.021	68.308
Industrial mining 2014	-44.258	13.146	70.379	-270.188	-80.927	102.812
Industrial mining 2015	-40.908	23.677	81.566	-113.630	57.901	240.359
$\text{LUC}^{Agriculture,Grassland}$	-57.318	**-45.508**	-33.347	-201.530	**-154.937**	-109.704
$\text{LUC}^{Agriculture,Pasture}$	3.570	**7.992**	12.204	-36.975	**-21.964**	-6.835
$\text{LUC}^{Grassland,Agriculture}$	31.102	**41.361**	51.270	94.670	**131.841**	170.244
$\text{LUC}^{Grassland,Pasture}$	22.064	**27.734**	33.221	-16.854	2.681	20.755
$\text{LUC}^{Pasture,Agriculture}$	7.769	**11.455**	15.042	5.117	**17.353**	29.922
$\text{LUC}^{Pasture,Grassland}$	3.729	**9.617**	15.443	6.406	**24.513**	42.381
Initial agriculture	-3.046	72.078	141.242	-280.961	**-158.098**	-37.280
Initial natural forest	843.526	**927.628**	1006.415	133.914	**292.139**	455.022
Initial forest plantation	-164.802	23.195	230.316	-2028.685	**-1537.833**	-1020.512
Initial grassland	-78.236	69.795	205.073	-824.289	**-440.438**	-55.053
Initial pasture	-106.356	-51.634	1.636	110.107	**195.257**	276.792
GDP growth	-2.072	-0.983	0.074	-1.310	2.739	7.002
Precipitation	-58.012	-10.584	38.025	-16.352	34.778	88.072
Elevation	-0.210	**-0.140**	-0.064	-0.239	**-0.155**	-0.065
ρ	0.542	**0.548**	0.562			
Observations	33,836					

Table S9: **Average direct and spillover effect estimates on forest loss (absolute) with yearly industrial mining effects.** Panel-structure (2005-2020) spatial Durbin model including time fixed effects. Dependent variable is annual forest loss in absolute ha. Estimates printed in bold type are statistically different from zero based on the 95 percent posterior credible interval. PM denotes posterior mean. Time-specific intercepts were excluded for more concise summary tables.

	Avg. direct effect			Avg. spillover		
Variables	2.5%	PM	97.5%	2.5%	PM	97.5%
Industrial mining × pre 2010	-3.363	24.273	51.907	-70.991	22.925	109.817
Industrial mining × since 2010	-16.146	8.422	35.816	-214.259	**-134.672**	-52.101
$LUC^{Agriculture,Grassland}$	-57.568	**-44.840**	-31.622	-198.658	**-154.720**	-109.298
$LUC^{Agriculture,Pasture}$	4.006	**8.157**	12.673	-36.427	**-21.429**	-7.261
$LUC^{Grassland,Agriculture}$	31.665	**41.430**	51.988	95.483	**132.325**	168.148
$LUC^{Grassland,Pasture}$	22.105	**27.808**	33.475	-15.260	2.677	20.191
$LUC^{Pasture,Agriculture}$	7.521	**11.351**	15.120	5.538	**17.135**	29.958
$LUC^{Pasture,Grassland}$	3.745	**9.605**	15.630	7.134	**24.251**	41.967
Initial agriculture	0.050	**71.828**	146.492	-283.760	**-155.334**	-38.692
Initial natural forest	848.623	**928.267**	1009.292	140.424	**295.186**	456.022
Initial forest plantation	-170.853	15.329	212.721	-2054.333	**-1543.981**	-1033.266
Initial grassland	-66.754	65.898	214.574	-801.754	**-442.679**	-106.585
Initial pasture	-109.089	-53.177	3.222	110.413	**194.838**	279.432
GDP growth	-2.033	-0.933	0.201	-1.421	2.873	6.979
Precipitation	-60.132	-9.785	38.744	-19.737	33.496	90.760
Elevation	-0.207	**-0.141**	-0.071	-0.234	**-0.152**	-0.064
ρ	0.542	**0.550**	0.562			
Observations	33,836					

Table S10: **Average direct and spillover effect estimates on forest loss (absolute) with pooled industrial mining effects.** Panel-structure (2005-2020) spatial Durbin model including time fixed effects. Dependent variable is annual forest loss in absolute ha. Estimates printed in bold type are statistically different from zero based on the 95 percent posterior credible interval. PM denotes posterior mean. Time-specific intercepts were excluded for more concise summary tables.

Variables	Avg. direct effect 2.5%	PM	97.5%	Avg. spillover 2.5%	PM	97.5%
Garimpo mining 2005	-0.207	0.458	1.176	-0.970	0.503	1.932
Garimpo mining 2006	-0.132	0.600	1.345	-0.093	1.406	2.801
Garimpo mining 2007	-0.193	0.532	1.285	0.757	**2.109**	3.585
Garimpo mining 2008	-0.189	0.524	1.216	0.654	**2.079**	3.487
Garimpo mining 2009	0.036	**0.729**	1.432	0.947	**2.288**	3.628
Garimpo mining 2010	-0.791	-0.027	0.683	-0.767	0.663	1.994
Garimpo mining 2011	-0.862	-0.142	0.582	-1.702	-0.371	1.009
Garimpo mining 2012	-0.791	-0.088	0.636	-1.780	-0.357	1.063
Garimpo mining 2013	-0.562	0.147	0.841	-2.130	-0.731	0.605
Garimpo mining 2014	-0.431	0.294	0.995	-1.989	-0.481	0.982
Garimpo mining 2015	-0.391	0.254	0.992	-1.180	0.236	1.570
$\text{LUC}^{Agriculture,ForestPlantation}$	0.085	**0.200**	0.311	-0.209	0.062	0.339
$\text{LUC}^{Agriculture,Grassland}$	0.025	**0.151**	0.289	-0.129	0.185	0.491
$\text{LUC}^{Agriculture,Pasture}$	-0.070	-0.022	0.027	0.030	**0.131**	0.234
$\text{LUC}^{NaturalForest,Agriculture}$	-0.088	0.033	0.150	-0.089	0.195	0.492
$\text{LUC}^{NaturalForest,ForestPlantation}$	0.002	**0.069**	0.137	-0.270	-0.131	0.004
$\text{LUC}^{NaturalForest,Grassland}$	-0.177	**-0.093**	-0.006	-0.084	0.126	0.342
$\text{LUC}^{NaturalForest,Pasture}$	-0.095	-0.037	0.022	-0.137	-0.047	0.044
$\text{LUC}^{ForestPlantation,Agriculture}$	0.026	**0.170**	0.308	-0.604	-0.185	0.218
$\text{LUC}^{ForestPlantation,Grassland}$	0.008	**0.188**	0.358	-0.791	-0.375	0.037
$\text{LUC}^{ForestPlantation,Pasture}$	-0.112	-0.028	0.057	-0.163	0.032	0.220
$\text{LUC}^{Grassland,Agriculture}$	-0.077	0.025	0.125	-0.216	0.022	0.280
$\text{LUC}^{Grassland,ForestPlantation}$	-0.133	-0.017	0.108	-0.532	**-0.273**	-0.003
$\text{LUC}^{Grassland,Pasture}$	-0.020	0.043	0.105	0.133	**0.257**	0.380
$\text{LUC}^{Pasture,Agriculture}$	-0.016	0.026	0.067	-0.077	0.015	0.106
$\text{LUC}^{Pasture,ForestPlantation}$	-0.010	0.046	0.102	-0.128	-0.018	0.097
$\text{LUC}^{Pasture,Grassland}$	-0.007	0.057	0.119	-0.141	-0.010	0.112
Initial agriculture	1.961	**2.846**	3.735	-3.449	**-2.293**	-1.057
Initial natural forest	-0.014	0.746	1.576	-0.691	0.535	1.664
Initial forest plantation	-2.036	0.206	2.339	-11.802	**-7.775**	-3.782
Initial grassland	-2.012	-0.576	0.929	-2.313	0.190	2.735
Initial pasture	-0.803	-0.181	0.481	-2.127	**-1.328**	-0.553
Initial income	-3.170	**-2.992**	-2.825	1.801	**2.161**	2.502
Human capital	3.155	**4.043**	4.903	-4.391	**-3.270**	-2.001
Population growth	-0.440	**-0.400**	-0.361	-0.153	-0.069	0.014
Population density	-0.655	0.089	0.880	1.254	**3.021**	4.744
GVA agriculture	-0.158	-0.065	0.022	-0.281	-0.119	0.031
GVA industry	-0.446	**-0.348**	-0.247	-0.654	**-0.474**	-0.287
GVA services	0.452	**0.561**	0.677	-0.017	0.225	0.454
Precipitation	-0.466	0.017	0.555	-0.209	0.340	0.850
Elevation	-0.001	0.000	0.001	-0.001	0.000	0.001
ρ	0.244	**0.257**	0.274			
Observations	23,727					

Table S11: **Average direct and spillover effect estimates on economic growth with yearly garimpo mining effects.** Panel-structure (2005-2020) spatial Durbin model including time fixed effects. Dependent variable is 5-year average annual GDP per capita growth rate. Estimates printed in bold type are statistically different from zero based on the 95 percent posterior credible interval. PM denotes posterior mean. Time-specific intercepts were excluded for more concise summary tables.

Variables	Avg. direct effect 2.5%	PM	97.5%	Avg. spillover 2.5%	PM	97.5%
Garimpo mining × pre 2010	0.268	**0.596**	0.921	1.164	**1.840**	2.583
Garimpo mining × since 2010	-0.228	0.068	0.383	-0.877	-0.209	0.467
$LUC^{Agriculture,ForestPlantation}$	0.089	**0.202**	0.314	-0.190	0.103	0.383
$LUC^{Agriculture,Grassland}$	0.017	**0.153**	0.283	-0.141	0.195	0.523
$LUC^{Agriculture,Pasture}$	-0.070	-0.020	0.029	0.027	**0.132**	0.237
$LUC^{NaturalForest,Agriculture}$	-0.078	0.038	0.152	-0.086	0.213	0.502
$LUC^{NaturalForest,ForestPlantation}$	0.003	**0.069**	0.140	-0.271	-0.131	0.021
$LUC^{NaturalForest,Grassland}$	-0.178	**-0.090**	-0.013	-0.086	0.121	0.349
$LUC^{NaturalForest,Pasture}$	-0.091	-0.038	0.015	-0.138	-0.053	0.032
$LUC^{ForestPlantation,Agriculture}$	0.014	**0.169**	0.319	-0.654	-0.221	0.206
$LUC^{ForestPlantation,Grassland}$	0.025	**0.191**	0.339	-0.811	-0.383	0.076
$LUC^{ForestPlantation,Pasture}$	-0.118	-0.029	0.056	-0.186	0.027	0.242
$LUC^{Grassland,Agriculture}$	-0.076	0.028	0.134	-0.227	0.041	0.288
$LUC^{Grassland,ForestPlantation}$	-0.141	-0.016	0.106	-0.564	**-0.296**	-0.019
$LUC^{Grassland,Pasture}$	-0.022	0.044	0.115	0.146	**0.274**	0.397
$LUC^{Pasture,Agriculture}$	-0.018	0.024	0.064	-0.077	0.019	0.116
$LUC^{Pasture,ForestPlantation}$	-0.014	0.046	0.104	-0.135	-0.017	0.103
$LUC^{Pasture,Grassland}$	-0.002	0.059	0.123	-0.120	0.002	0.122
Initial agriculture	1.982	**2.809**	3.696	-3.403	**-2.206**	-0.995
Initial natural forest	-0.089	0.747	1.548	-0.621	0.629	1.926
Initial forest plantation	-2.256	0.049	2.382	-12.661	**-8.193**	-3.655
Initial grassland	-2.015	-0.561	0.909	-2.682	0.043	2.595
Initial pasture	-0.799	-0.179	0.458	-2.216	**-1.437**	-0.676
Initial income	-3.137	**-2.966**	-2.782	1.742	**2.073**	2.421
Human capital	3.120	**4.037**	4.851	-4.448	**-3.213**	-2.016
Population growth	-0.441	**-0.403**	-0.363	-0.200	**-0.110**	-0.018
Population density	-0.700	0.151	1.042	1.407	**3.201**	5.120
GVA agriculture	-0.154	-0.065	0.023	-0.304	-0.138	0.020
GVA industry	-0.438	**-0.353**	-0.265	-0.740	**-0.539**	-0.334
GVA services	0.457	**0.562**	0.667	0.035	**0.297**	0.553
Precipitation	-0.438	0.036	0.498	-0.151	0.345	0.851
Elevation	-0.001	0.000	0.001	-0.001	0.000	0.001
ρ	0.244	**0.257**	0.274			
Observations	23,727					

Table S12: **Average direct and spillover effect estimates on economic growth with pooled garimpo mining effects.** Panel-structure (2005-2020) spatial Durbin model including time fixed effects. Dependent variable is 5-year average annual GDP per capita growth rate. Estimates printed in bold type are statistically different from zero based on the 95 percent posterior credible interval. PM denotes posterior mean. Time-specific intercepts were excluded for more concise summary tables.

Variables	Avg. direct effect 2.5%	PM	97.5%	Avg. spillover 2.5%	PM	97.5%
Garimpo mining 2005	0.035	**0.073**	0.110	0.258	**0.405**	0.561
Garimpo mining 2006	0.024	**0.063**	0.102	0.275	**0.421**	0.567
Garimpo mining 2007	0.012	**0.049**	0.085	0.219	**0.362**	0.506
Garimpo mining 2008	-0.036	0.001	0.038	0.003	**0.148**	0.285
Garimpo mining 2009	-0.014	0.023	0.062	-0.170	-0.033	0.098
Garimpo mining 2010	-0.056	-0.018	0.019	-0.165	-0.041	0.091
Garimpo mining 2011	-0.052	-0.017	0.023	-0.300	**-0.165**	-0.020
Garimpo mining 2012	-0.048	-0.008	0.032	-0.276	-0.139	0.001
Garimpo mining 2013	-0.057	-0.022	0.016	-0.088	0.042	0.169
Garimpo mining 2014	-0.038	-0.002	0.035	-0.021	0.116	0.249
Garimpo mining 2015	-0.031	0.005	0.043	0.019	**0.146**	0.282
$LUC^{Agriculture,Grassland}$	-0.018	**-0.010**	-0.003	-0.065	**-0.033**	-0.001
$LUC^{Agriculture,Pasture}$	-0.006	**-0.004**	-0.001	-0.031	**-0.021**	-0.011
$LUC^{Grassland,Agriculture}$	-0.008	-0.002	0.004	-0.017	0.008	0.031
$LUC^{Grassland,Pasture}$	-0.006	-0.003	0.000	-0.024	-0.011	0.001
$LUC^{Pasture,Agriculture}$	0.001	**0.003**	0.005	0.007	**0.015**	0.025
$LUC^{Pasture,Grassland}$	-0.005	-0.002	0.001	0.003	**0.015**	0.028
Initial agriculture	-0.016	0.027	0.069	-0.256	**-0.179**	-0.107
Initial natural forest	0.359	**0.398**	0.434	-0.248	**-0.156**	-0.059
Initial forest plantation	0.087	**0.192**	0.297	-0.905	**-0.603**	-0.293
Initial grassland	-0.004	0.067	0.137	-0.172	0.044	0.251
Initial pasture	0.025	**0.052**	0.081	-0.092	-0.044	0.006
GDP growth	-0.001	-0.000	0.001	0.001	**0.004**	0.007
Precipitation	-0.039	-0.016	0.007	0.041	**0.067**	0.093
Elevation	-0.000	**-0.000**	-0.000	-0.000	**-0.000**	-0.000
ρ	0.642	**0.650**	0.662			
Observations	23,727					

Table S13: **Average direct and spillover effect estimates on forest loss (relative) with yearly garimpo mining effects.** Panel-structure (2005-2020) spatial Durbin model including time fixed effects. Dependent variable is annual forest loss in ha per km^2. Estimates printed in bold type are statistically different from zero based on the 95 percent posterior credible interval. PM denotes posterior mean. Time-specific intercepts were excluded for more concise summary tables.

Variables	Avg. direct effect 2.5%	PM	97.5%	Avg. spillover 2.5%	PM	97.5%
Garimpo mining $\times$ pre 2010	0.026	**0.044**	0.061	0.220	**0.290**	0.364
Garimpo mining $\times$ since 2010	-0.026	-0.010	0.005	-0.077	-0.009	0.061
$LUC^{Agriculture,Grassland}$	-0.017	**-0.010**	-0.003	-0.068	-0.034	0.001
$LUC^{Agriculture,Pasture}$	-0.006	**-0.004**	-0.001	-0.033	**-0.022**	-0.012
$LUC^{Grassland,Agriculture}$	-0.008	-0.003	0.003	-0.020	0.008	0.034
$LUC^{Grassland,Pasture}$	-0.007	-0.003	0.000	-0.026	-0.012	0.002
$LUC^{Pasture,Agriculture}$	0.001	**0.003**	0.005	0.007	**0.016**	0.026
$LUC^{Pasture,Grassland}$	-0.005	-0.002	0.001	0.004	**0.017**	0.030
Initial agriculture	-0.017	0.025	0.068	-0.275	**-0.193**	-0.112
Initial natural forest	0.362	**0.399**	0.436	-0.218	**-0.124**	-0.028
Initial forest plantation	0.082	**0.187**	0.302	-1.006	**-0.648**	-0.294
Initial grassland	-0.007	0.065	0.142	-0.172	0.043	0.250
Initial pasture	0.023	**0.053**	0.082	-0.099	-0.044	0.011
GDP growth	-0.001	-0.000	0.001	0.001	**0.005**	0.008
Precipitation	-0.037	-0.014	0.008	0.043	**0.069**	0.095
Elevation	-0.000	**-0.000**	-0.000	-0.000	**-0.000**	-0.000
ρ	0.642	**0.652**	0.662			
Observations	23,727					

Table S14: **Average direct and spillover effect estimates on forest loss (relative) with pooled garimpo mining effects.** Panel-structure (2005-2020) spatial Durbin model including time fixed effects. Dependent variable is annual forest loss in ha per km^2. Estimates printed in bold type are statistically different from zero based on the 95 percent posterior credible interval. PM denotes posterior mean. Time-specific intercepts were excluded for more concise summary tables.

Variables	Avg. direct effect 2.5%	PM	97.5%	Avg. spillover 2.5%	PM	97.5%
Garimpo mining 2005	549.497	**675.464**	812.319	1975.496	**2288.662**	2590.349
Garimpo mining 2006	270.032	**394.822**	519.557	1788.484	**2108.256**	2407.248
Garimpo mining 2007	200.639	**321.681**	456.241	1006.397	**1306.967**	1608.615
Garimpo mining 2008	95.080	**226.289**	347.738	685.723	**984.721**	1279.580
Garimpo mining 2009	-3.208	117.198	244.982	-85.157	222.462	527.156
Garimpo mining 2010	-82.254	40.964	158.689	-214.486	101.563	418.058
Garimpo mining 2011	-25.114	99.217	224.476	-165.303	119.297	382.568
Garimpo mining 2012	-60.261	62.327	192.055	-320.934	-30.521	254.498
Garimpo mining 2013	18.402	**150.603**	275.145	9.682	**300.872**	594.216
Garimpo mining 2014	-117.352	16.789	145.094	121.055	**397.356**	676.172
Garimpo mining 2015	-17.924	110.076	230.983	364.957	**649.015**	934.871
$LUC^{Agriculture,Grassland}$	-81.134	**-58.827**	-37.766	-152.229	**-83.653**	-13.655
$LUC^{Agriculture,Pasture}$	2.053	**10.419**	18.801	-67.470	**-45.241**	-25.114
$LUC^{Grassland,Agriculture}$	12.568	**29.511**	46.388	56.988	**111.873**	160.876
$LUC^{Grassland,Pasture}$	18.989	**29.679**	40.754	-33.211	-6.666	19.981
$LUC^{Pasture,Agriculture}$	23.379	**30.853**	38.001	37.531	**56.052**	72.977
$LUC^{Pasture,Grassland}$	-0.157	10.277	20.551	35.441	**60.800**	85.292
Initial agriculture	86.320	**230.882**	373.377	-260.520	-52.806	142.266
Initial natural forest	1685.359	**1817.029**	1961.441	-1524.171	**-1304.452**	-1080.541
Initial forest plantation	-378.967	-20.176	374.037	-1519.775	**-785.946**	-62.787
Initial grassland	133.134	**376.916**	622.041	-1226.866	**-745.074**	-282.584
Initial pasture	-293.186	**-196.111**	-94.971	396.229	**528.159**	653.015
GDP growth	-4.068	-1.931	0.253	1.867	**8.322**	14.388
Precipitation	-131.431	-49.900	31.239	57.175	**141.322**	225.985
Elevation	-0.594	**-0.471**	-0.354	0.038	**0.176**	0.327
ρ	0.403	**0.418**	0.433			
Observations	23,727					

Table S15: **Average direct and spillover effect estimates on forest loss (absolute) with yearly garimpo mining effects.** Panel-structure (2005-2020) spatial Durbin model including time fixed effects. Dependent variable is annual forest loss in absolute ha. Estimates printed in bold type are statistically different from zero based on the 95 percent posterior credible interval. PM denotes posterior mean. Time-specific intercepts were excluded for more concise summary tables.

	Avg. direct effect			Avg. spillover		
Variables	2.5%	PM	97.5%	2.5%	PM	97.5%
Garimpo mining × pre 2010	310.914	**368.772**	426.497	1397.247	**1564.523**	1724.847
Garimpo mining × since 2010	33.241	**84.233**	134.768	155.644	**300.739**	449.400
$\text{LUC}^{Agriculture,Grassland}$	-81.582	**-57.297**	-35.794	-157.779	**-84.997**	-11.675
$\text{LUC}^{Agriculture,Pasture}$	2.002	**10.507**	18.731	-65.995	**-42.966**	-20.811
$\text{LUC}^{Grassland,Agriculture}$	10.681	**29.011**	48.737	59.182	**115.156**	172.455
$\text{LUC}^{Grassland,Pasture}$	19.039	**30.278**	40.953	-26.748	0.780	28.781
$\text{LUC}^{Pasture,Agriculture}$	23.986	**31.559**	39.105	39.963	**60.855**	82.475
$\text{LUC}^{Pasture,Grassland}$	0.681	**10.903**	21.335	37.902	**64.443**	93.230
Initial agriculture	96.797	**229.492**	377.394	-239.106	-29.769	171.852
Initial natural forest	1684.967	**1809.217**	1950.444	-1465.802	**-1227.971**	-1000.397
Initial forest plantation	-399.543	-38.782	330.380	-1689.012	**-899.341**	-92.575
Initial grassland	114.876	**362.051**	607.162	-1261.282	**-763.853**	-282.395
Initial pasture	-288.524	**-190.240**	-87.414	416.022	**552.229**	698.001
GDP growth	-4.399	-2.055	0.246	1.188	**7.592**	14.460
Precipitation	-121.101	-44.920	40.429	56.220	**145.489**	230.938
Elevation	-0.593	**-0.469**	-0.355	-0.004	0.139	0.290
ρ	0.413	**0.427**	0.443			
Observations	23,727					

Table S16: **Average direct and spillover effect estimates on forest loss (absolute) with pooled garimpo mining effects.** Panel-structure (2005-2020) spatial Durbin model including time fixed effects. Dependent variable is annual forest loss in absolute ha. Estimates printed in bold type are statistically different from zero based on the 95 percent posterior credible interval. PM denotes posterior mean. Time-specific intercepts were excluded for more concise summary tables.

References

1. Manzolli, B. *et al. The Prevalence of Illegal Gold Production in Brazil* (Editora IGC/UFMG, Belo Horizonte, 2021).
2. Cozendey, G., Pereira, L., Pucci, R. & Chiavari, J. *Presidential Decrees Exacerbate the Contradiction in Mining Regulations at the Expense of the Environment.* (Climate Policy Initiative, Rio de Janeiro, 2022).
3. MapBiomas. *A expansão da mineração e do garimpo no Brasil nos últimos 36 anos. Destaques do Mapeamento Anual de Mineração e Garimpo no Brasil entre 1985 a 2020* https://acervo.socioambiental.org/sites/default/files/documents/prov0380.pdf. Accessed: 13 Sep 2023 (2021).
4. Maus, V. *et al.* An update on global mining land use. *Scientific Data* **9,** 1–11 (2022).
5. Anselin, L. *Spatial Econometrics: Methods and Models* (Springer Science & Business Media, Berlin, 2013).
6. Pebesma, E. Simple features for R: standardized support for spatial vector data. *R J.* **10,** 439–446 (2018).
7. Bivand, R. S., Pebesma, E. & Gómez-Rubio, V. *Applied Spatial Data Analysis with R, Second edition* https://asdar-book.org/ (Springer, NY, 2013).
8. R Core Team. *R: A Language and Environment for Statistical Computing* R Foundation for Statistical Computing (Vienna, Austria, 2024).
9. Plümper, T. & Neumayer, E. Model specification in the analysis of spatial dependence. *Eur. J. Political Res.* **49,** 418–442 (2010).
10. LeSage, J. P. & Pace, R. K. *Introduction to Spatial Econometrics* (Taylor & Francis, Boca Raton, 2009).
11. Kuschnig, N. Bayesian spatial econometrics: a software architecture. *J. Spat. Econ.* **3,** 1–25 (2022).
12. LeSage, J. & Parent, O. Bayesian model averaging for spatial econometric models. *Geogr. Anal.* **39,** 241–267 (2007).
13. Ritter, C. & Tanner, M. Facilitating the Gibbs sampler: The Gibbs stopper and the Griddy-Gibbs sampler. *J. Am. Stat. Assoc.* **87,** 861–868 (1992).
14. Geweke, J. *Evaluating the accuracy of sampling-based approaches to the calculations of posterior moments* in *Bayesian Statistics* (eds Bernardo, J., Berger, J., Dawid, A. & Smith, A.) 167–193 (Oxford Univ., Oxford, 1992).
15. Plummer, M., Best, N., Cowles, K. & Vines, K. CODA: Convergence diagnosis and output analysis for MCMC. *R News* **6,** 7–11 (2006).
16. López-Bazo, E., Vayá, E. & Artís, M. Regional externalities and growth: evidence from European regions. *J. Reg. Sci.* **44,** 43–73 (2004).

17. Resende, G. M., de Carvalho, A. X. Y., Sakowski, P. A. M. & Cravo, T. A. Evaluating multiple spatial dimensions of economic growth in Brazil using spatial panel data models. *Ann. Regional Sci.* **56,** 1–31 (2016).

18. LeSage, J. P. & Fischer, M. M. Spatial growth regressions: model specification, estimation and interpretation. *Spat. Econ. Anal.* **3,** 275–304 (2008).

19. MapBiomas. *MapBiomas Project – Collection 8.0 of the Annual Land Use Land Cover Maps of Brazil,* accessed on 7 Sep 2023 through the link: https://brasil.mapbiomas.org/estatisticas/, released under a CC Attribution-ShareAlike 4.0 International Licence (CC BY-SA 4.0).

20. IBGE. *Instituto Brasileiro de Geografia e Estatística, Malha Municipal* https://www.ibge.gov.br/geociencias/organizacao-do-territorio/malhas-territoriais/15774-malhas.html?=&t=sobre. Accessed: 6 Sep 2023 (Rio de Janeiro, 2023).

21. Pereira, R. H. M. & Goncalves, C. N. *geobr: Download Official Spatial Data Sets of Brazil* R package version 1.7.0 (2022).

22. IBGE. *Instituto Brasileiro de Geografia e Estatística, Estimativas de Populacao* https://ftp.ibge.gov.br/Estimativas_de_Populacao/. Accessed: 4 Sep 2023 (Rio de Janeiro, 2023).

23. IBGE. *Instituto Brasileiro de Geografia e Estatística, PIB municipios* https://ftp.ibge.gov.br/Pib_Municipios/. Accessed: 10 Aug 2023 (Rio de Janeiro, 2023).

24. FIRJAN. *Education index (IFDM Educação) Version 2018* https://www.firjan.com.br/ifdm/downloads/. Accessed: 6 Sep 2023 (2018).

25. CRU. *Climatic Research Unit (CRU) Time-Series (TS) Version 3.21 of High Resolution Gridded Data of Month-by-month Variation in Climate* http://dx.doi.org/10.5285/D0E1585D-3417-485F-87AE-4FCECF10A992. Accessed: 3 Aug 2023 (2021).

26. USGS. *Global Multi-resolution Terrain Elevation Data GMTED2010* https://www.usgs.gov/core-science-systems/eros/coastal-changes-and-impacts/gmted2010. Accessed: 3 Aug 2023 (2021).

27. Aragón, F. M. & Rud, J. P. Natural resources and local communities: evidence from a Peruvian gold mine. *Am. Econ. J. Econ. Policy* **5,** 1–25 (2013).

28. Arias, M., Atienza, M. & Cademartori, J. Large mining enterprises and regional development in Chile: between the enclave and cluster. *J. Econ. Geogr.* **14,** 73–95 (2013).

29. Aragón, F. M., Chuhan-Pole, P. & Land, B. C. The local economic impacts of resource abundance: What have we learned? *World Bank Policy Research Working Paper No. 7263* (2015).

30. Auty, R. M. *Sustaining Development in Mineral Economies: The Resource Curse Thesis* (Routledge, New York, 1993).

31. Sachs, J. D. & Warner, A. M. The curse of natural resources. *Eur. Econ. Rev.* **45,** 827–838 (2001).

32. Humphreys, M., Sachs, J. D. & Stiglitz, J. E. *Escaping the Resource Curse* (Columbia Univ., New York, 2007).

33. Manzano, O. & Gutiérrez, J. D. The subnational resource curse: theory and evidence. *Extr. Ind. Soc.* **6,** 261–266 (2019).

34. Owen, J., Kemp, D. & Marais, L. The cost of mining benefits: localising the resource curse hypothesis. *Resour. Policy* **74,** 102289 (2021).

35. Lo, M. G. *et al.* Nickel mining reduced forest cover in Indonesia but had mixed outcomes for well-being. *One Earth* **7,** 2019–2033 (2024).

36. Solow, R. M. A contribution to the theory of economic growth. *Q. J. Econ.* **70,** 65–94 (1956).

37. Lucas, R. E. On the mechanics of economic development. *J. Monet. Econ.* **22,** 3–42 (1988).

38. Romer, P. M. Endogenous technological change. *J. Political Econ.* **98,** S71–S102 (1990).

39. Giljum, S. *et al.* A pantropical assessment of deforestation caused by industrial mining. *PNAS* **119,** e2118273119 (2022).

40. Sonter, L. J. *et al.* Mining drives extensive deforestation in the Brazilian Amazon. *Nat. Commun.* **8,** 1013 (2017).

41. Busch, J. & Ferretti-Gallon, K. What drives deforestation and what stops it? A meta-analysis. *Rev. Environ. Econ. Policy* **11,** 3–23 (2017).

42. Kuschnig, N., Crespo Cuaresma, J., Krisztin, T. & Giljum, S. Spatial spillover effects from agriculture drive deforestation in Mato Grosso, Brazil. *Sci. Rep.* **11,** 21804 (2021).
